# Enzyme stabilisation due to incorporation of a fluorinated non-natural amino acid at the protein surface

**DOI:** 10.1038/s41598-024-79711-6

**Published:** 2024-11-14

**Authors:** Arka Mukhopadhyay, Yiwen Li, Matthew J. Cliff, Alexander P. Golovanov, Paul A. Dalby

**Affiliations:** 1 Department of Biochemical Engineering, University College London, Gordon Street, London, WC1H 0AH UK; 2 Manchester Institute of Biotechnology, 131 Princess St, Manchester, M1 7DN UK; 3 Department of Chemistry, School of Natural Sciences, Faculty of Science and Engineering, The University of Manchester, M1 7DN Manchester, UK

**Keywords:** Molecular modelling, NMR spectroscopy, Biophysical chemistry, Enzymes

## Abstract

We have previously engineered *E. coli* transketolase (TK) enzyme variants that accept new substrates such as aliphatic or aromatic aldehydes, and also with improved thermal stability. Irreversible aggregation is the primary mechanism of deactivation for TK in the buffers used for biocatalysis, and so we were interested in determining the extent to which this remains true in more complex media, crude cell lysates or even in vivo. Such understanding would better guide future protein engineering efforts. NMR offers a potential approach to probe protein structure changes, aggregation, and diffusion, and^19^F-labelled amino acids are a useful NMR probe for complex systems with little or no background signal from the rest of the protein or their environment. Here we labelled *E. coli* TK with two different^19^F probes, trifluoromethyl-L-phenylalanine (tfm-Phe), and 4-fluoro phenylalanine (4 F-Phe), through site specific non-natural amino acid incorporation. We targeted them to residue K316, a highly solvent exposed site located at the furthest point from the enzyme active sites. Characterisation of the^19^F-labelled TK variants revealed surprising effects of these mutations on stability, and to some extent on activity. While variant TK-tfm-Phe led to a 7.5 °C increase in the thermal transition midpoint (*T*_m_) for denaturation, the TK-4 F-Phe variant largely abolished the aggregation of the enzyme when incubated at 50 °C^19^. F-NMR revealed different behaviours in response to temperature increases for the two TK variants, displaying opposite temperature gradient chemical shifts, and diverging motion regimes, suggesting that the mutations affected differently both the local environment at this site, and its temperature-induced dynamics. A similar incubation of TK at 40–55 °C is also known to induce higher cofactor-binding affinities, leading to an apparent heat activation under low cofactor concentration conditions. We have hypothesised previously that a heat-inducible conformational change in TK leads to this effect^1^. H-NMR revealed a temperature-dependent re-structuring of methyl groups, also at 30–50 °C, which may be linked to the heat activation. While our kinetic studies were not expected to observe the heat activation event due to the high cofactor concentrations used, this was not the case for TK-4 F-Phe, which did appear to heat activate slightly at 45 °C. This implied that the mutations at K316 could influence cofactor-binding, despite their location at 47 Å from either active site. Such long-distance effects of mutations are not unprecedented, and indeed we have previously shown how distant mutations can influence active-site loop stability and function in TK, mediated via dynamically coupled networks of residues. Molecular dynamics simulations of the two^19^F containing variants similarly revealed networks of residues that could couple the changes in dynamics at residue K316, through to changes in active site dynamics. These results independently highlight the sensitivity of active-site function to distant mutations coupled through correlated dynamic networks of residues. They also highlight the potential influence of surface-incorporated probes on protein stability and function, and the need to characterise them well prior to further studies.

**Supplementary Information:**

The online version contains supplementary material available at 10.1038/s41598-024-79711-6.

## Introduction

Enzymes used as industrial biocatalysts often need to function at elevated temperatures, and maintain stability for long reaction durations. Higher temperatures enhance reaction rates, increase reactant solubility, and lower the risk of contamination in biocatalytic reactors^1,2^. Such conditions require high thermostability and low aggregation propensity, which is therefore often a target for protein engineering^3,4^.

Transketolase (TK) has immense potential for the regio- and stereo-specific synthesis of C-C bonds in complex carbohydrates and other high-value compounds^5,6^. TK is a thiamine diphosphate-dependent (ThDP) enzyme, that catalyses the reversible transfer of a C2-ketol unit from D-xylulose- 5-phosphate to either D-ribose-5-phosphate or D-erythrose-4- phosphate in living cells^7,8^. It is a key enzyme of the pentose phosphate pathway (PPP), ubiquitous in all organisms, and provides a unique link between glycolysis and the non-oxidative phase of the PPP. For biocatalysis, TK can reversibly transfer a two-carbon ketol group from a simpler donor substrate β-hydroxypyruvate (β-HPA) to an acceptor aldehyde, releasing CO_2_ to drive the reaction to completion. Homodimeric TK consists of two 73–74 kDa monomers, each comprised of a pyrophosphate (PP)-binding domain (residues 1–322), pyrimidine (Pyr)-binding domain (residues 323–539) and a C-terminal domain (residues 540–663)^2^. Two cofactors, thiamine (ThDP) and a Mg^2+^ (or Ca^2+^) ion, bind into each active site formed at the two identical interfaces between the PP and Pyr domains of opposite subunits.

Transketolases have been engineered for activity and substrate shifts, including to accept aromatic aldehydes, pyruvate as an alternative donor, and even both simultaneously^9–11^. Meanwhile, the engineering of stability has also been critical for *E. coli* TK, to enable the enzyme to operate at elevated temperatures^12^, but also to increase the potential to absorb mutations that alter catalytic function^13^. Our foundational approach to enzyme engineering of TK has been to employ site-directed saturation mutagenesis. Such approaches have been typically guided by structure, bioinformatics, substrate docking or molecular dynamics simulations^3,13–16^, but also from biophysical studies of enzyme stability^1,15,17^. While this strategy relies on gaining an understanding of structure and function through different approaches to inform predictions^4,18^, unlike classical directed evolution, it also allows for significant improvements to be made with efficient smaller libraries^19,20^. With this in mind, we set out to gain a deeper understanding of the stability limitations for TK to guide protein engineering. TK has a broad optimum activity at 20–40 °C and loses activity rapidly at above 55 °C due to irreversible aggregation in simple buffer systems^1^. However, overexpression of TK at 37 °C leads to two forms of the enzyme with high and low affinity for cofactors^21^. Incubation of TK at 40–55 °C also leads to an apparent increase in enzyme activity^1^. This was later characterised to be caused by a shift in population of the two forms of the enzyme after heat treatment, resulting in a net increase in affinity of the enzyme for the cofactors, and observed as an increase in activity at low cofactor concentrations^10,22^. The thermal stability landscape for TK is therefore complex, and so further protein engineering could be guided well by a better understanding of whether aggregation remains the dominant deactivation mechanism in the complex media often used in biocatalysis.

Site-specific incorporation of fluorine into proteins via ^19^F-labelled non-natural amino acids (nnAA) can assist with elucidation and dynamic characterization of structure in vivo and in vitro when combined with ^19^F-NMR spectroscopy^22^. Using an orthogonal tRNA/aminoacyl-tRNA synthetase pair a ^19^F-nnAA can be site-specifically incorporated with high efficiency^23^. The unique synthetase/tRNA pair functions with high translational efficiency and fidelity when incorporating trifluoromethyl-L-phenylalanine (tfm-Phe), 4-fluoro phenylalanine (4 F-Phe) into sites encoded as amber (TAG) codons in *E. coli*^23–27^. ^19^F NMR spectroscopy is highly sensitive to its surrounding environment, with 100% natural abundance, and can monitor protein movements due to covalent modification, ligand binding, or other molecular interactions^28^. ^19^F NMR has been widely used to investigate even large sized proteins, such as a 100 kDa citrate synthase and a 210 kDa trimeric pyruvate kinase^29,30^.

In this study we site-specifically incorporated tfm-Phe and 4 F-Phe at the same highly solvent exposed position in TK, replacing K316, and characterised their differential impact on both catalytic activity and stability, including an assessment of any impact on the known heat-activation effects. The temperature dependencies of enzyme kinetics, activity retention, and aggregation were determined to evaluate the impact of the incorporation of fluorinated non-natural amino acids on stability and function. ^19^F and ^1^H NMR spectroscopy was also used to monitor the effects of temperature and labelling on local structure.

While the enzyme kinetics were largely unchanged by the non-natural amino acid substitutions, with some small differences, the stabilities of the variants were markedly increased, with a 7.5 °C increase in the thermal transition midpoint (*T*_m_) for TK-tfm-Phe, and a near-complete elimination of aggregate formation at 50 °C for TK-4 F-Phe. Here, enzyme kinetics were carried out under high cofactor conditions where heat activation is no longer observable for WT or TK-tfm-Phe, and yet TK-4 F-Phe still demonstrated a small heat activation at 40–45 °C. ^19^F and ^1^H NMR spectroscopy revealed temperature dependent structural changes contemporaneous with the heat activation. Molecular dynamics simulations highlighted the potential networks of residues through which the ^19^F labels could exert an influence on active site structure and function.

## Materials and methodology

### Materials

All chemicals were obtained from Sigma-Aldrich (Merck, Germany) except non-natural amino acids trifluoromethyl-L-phenylalanine (tfm-Phe) and 4-fluoro-phenylalanine (4 F-Phe) obtained from ChemCruz (Texas, USA).

### Methods

#### Expression and purification of transketolase

WT transketolase (WT TK) was overexpressed at 37 °C as previously from *E. coli* XL-10-Gold was transformed with plasmid pQR791 harbouring the wild-type transketolase gene with an N-terminal His_6_ tag^15^. The TK gene was expressed as 2 L cell cultures centrifuged at 2000 g for 20 min. Cell paste was then resuspended in 50 mM Tris-HCl, pH 7.0, and the cells lysed by sonication (Soniprep150, Sanyo UK). TK was purified using a Ni-NTA Sepharose column (Qiagen) according to the manual. After confirming the purified protein size and purity from 12% SDS-PAGE, samples were buffer exchanged into 50 mM Tris-HCl, pH 7.0, and concentrated to 1 mg/ml using an Amicon Ultra 10 kDa centrifugal filter (Millipore) prior to storage at 4 °C. Protein concentrations were determined by absorbance at 280 nm in 6 M Guanidine-HCl and 20 mM Sodium Phosphate, pH 6.5 using a Nanodrop spectrophotometer and an extinction coefficient (ε) of 92,630 L mol^− 1^ cm^− 1^.

#### Incorporation of nnAAs into transketolase

Two different nnAAs, trifluoromethyl-L-phenylalanine (tfm-Phe) and 4-fluoro-phenylalanine (4 F-Phe), were incorporated site-specifically at the lysine (Lys) 316 position, as follows. The AAG codon for Lys316 of TK in pQR791 was site-specifically mutated to a TAG amber stop codon using the QuikChange kit (Agilent, USA) and the following primers.

Amber316Fwd: 5’ GAAATTCGCTGCTTACGCGTAGGCTTATCCGCAGGAAGCC 3’.

Amber316Rev: 5’ GGCTTCCTGCGGATAAGCCTACGCGTAAGCAGCGAATTTC 3’.

The nnAA incorporation was carried out by co-transformation of the amberless *E. coli* strain *C321.ΔA.exp* (Addgene #49018) with the *pUltra* plasmid (Addgene #48215) carrying the tfm-Phe/ 4 F-Phe specific aminoacyl synthetase gene, and the pQR791 plasmid carrying the TK gene containing the amber codon at residue 316. The mutated TK gene was then overexpressed for eight hours in the presence of 1 mM nnAA (tfm-Phe or 4 F-Phe) and 1 mM IPTG. Proteins were then purified and concentrated as above. Each nnAA incorporation was confirmed by LC-ESI-MS as described previously^10^ from 20 µL TK at 0.2 µg/µL injected onto an Agilent PLRP-S (150 mm × 2.1 mm, 1000 Å, 8 μm) column on an Agilent 1100/1200 LC system connected to a 6510 A QTOF mass spectrometer (Agilent, UK). Data were processed in MassHunter software (version B.07.00) and deconvolved using the maximum entropy deconvolution algorithm.

#### Holo-transketolase enzyme preparation

Purified apo-transketolase variants at 1 mg/ml in 50 mM Tris.HCl, pH 7.0 were mixed gently with cofactor solution (2.4 mM ThDP, 9 mM MgCl_2_, 50 mM Tris.HCl, pH 7.0) to obtain 0.15 mg/ml of holo-TK with 2.04 mM ThDP and 7.65 mM MgCl_2_ in 50 mM Tris.HCl, pH 7.0, and incubated for 15 min at 21 °C. These holo-TK preparations were used in activity assays, enzyme kinetics, SEC and DLS and NMR as described below.

#### NMR spectroscopy

Holo-TK samples were concentrated up to 15 mg/ml in 2.4 mM ThDP and 9 mM MgCl_2_, 50 mM Tris.HCl, pH 7.0. All 1D ^1^H- and ^19^F-NMR spectra were recorded for 0.22 mM protein solutions in aqueous buffer containing 10% D_2_O and 0.01–0.2% TSP, using a Bruker 500 MHz NMR AVIII spectrometer with a QCI-F cryoprobe equipped with z-gradients. Temperature ranges started from 5 °C until 50 °C in 5–10 °C increments. Proton spectra were recorded using excitation sculpting water suppression.

#### Initial velocities for enzyme reactions at various temperatures

Triplicate reactions were initiated in a 96-well microplate, by adding 100 µL holo-TK stocks (0.15 mg/ml TK, 2.04 mM ThDP, 7.65 mM MgCl_2_, 50 mM Tris.HCl, pH 7.0) into 50 µL pre-warmed substrate mix containing 150 mM GA and 150 mM HPA in water, for final substrate concentrations of 50 mM each, and 0.1 mg/ml holo-TK in 1.36 mM ThDP, 5.1 mM MgCl_2_, 33 mM Tris.HCl, pH 7.0. These were incubated at different temperatures (25–65 °C), and shaken at 300 rpm using a Thermomixer (Eppendorf, Germany). After various time intervals of 0 to 60 min, 10 µL of reaction mix was quenched with 190 µL 0.1% trifluoroacetic acid (TFA). Samples were subsequently analysed by HPLC (Dionex Ultimate, Thermofisher, UK) with a Bio-Rad Aminex HPX-87 H reverse phase column (300 × 7.8 mm^2^) (Bio-Rad Labs., Richmond, CA, USA), via Chromeleon client 6.60 software, to separate and analyse changes in the concentrations of substrate (GA) and product (Ery) during reactions, as described previously^22^. Initial velocities were determined from exponential fits to product formation over time, in OriginPro9.0, calculating the first derivative at t = 0, and then averaged for replicates.

#### Residual initial velocities at 21 °C after 15 min heat shocks at various temperatures

Heat shocks were set up in a 96-well microplate with 100 µL holo-TK stocks (0.15 mg/ml TK, 2.04 mM ThDP, 7.65 mM MgCl_2_, 50 mM Tris.HCl, pH 7.0) and incubated at different temperatures (25–65 °C) for 15 min with shaking at 300 rpm using a Thermomixer (Eppendorf, Germany), then cooled to 21 °C for 10 min prior to activity assays at 21 °C. Reactions were started with 50 µL pre-warmed substrate mix as above to give 50 mM GA, 50 mM HPA, 0.1 mg/ml holo-TK, 1.36 mM ThDP, 5.1 mM MgCl_2_, and 33 mM Tris.HCl, pH 7.0. After various time intervals of 0 to 60 min, 10 µL of reaction mix was quenched and analysed by HPLC as above to obtain residual initial velocities.

#### Enzyme kinetics

We determined initial velocities *K*_m_ (for GA), *V*_max_ and *k*_cat_ for all TK variants in the enzymatic reaction between saturating 50 mM HPA and varying (5–100 mM) GA, after pre-incubating the enzyme at either 21 ^o^C or 50 ^o^C for 15 min as above, and re-cooling to 21 °C for 10 min prior to activity assays at 21 °C. Reactions were started with 50 µL pre-warmed substrate mix to give 50 mM HPA, 5–100 mM GA, 0.1 mg/ml holo-TK, 1.36 mM ThDP, 5.1 mM MgCl_2_, and 33 mM Tris.HCl, pH 7.0. Aliquots of 10 µL were quenched at various times over 60 min by adding 190 µL of 0.1% (v/v) TFA, and analysed as described above. Up to six replicates from different enzyme preparations were obtained for each condition. All initial velocity data were fitted by non-linear regression to the Michaelis–Menten equation to determine the *K*_m_ (for GA), *V*_max_ and *k*_cat_ of wild-type TK and the variants (at 50 mM HPA), using software OriginPro9.0.

#### Dynamic light scattering

The WT TK samples both before and after heat treatments, were analysed by DLS on a Nano Zetasizer (Malvern, UK). Each scattering curve was obtained from 10 repeated scans, and each in triplicate at 20 °C.

#### SEC HPLC

The WT and variant TK samples above, both before and after heat treatments, were analysed on an HPLC instrument (1200 series, Agilent, UK), using a Zorbax-GF250 size-exclusion chromatography (SEC) column with 1 mL/min flow rate of 200 mM sodium phosphate pH 7.0 as the mobile phase, with the column at room temperature. Elution profiles at 280 nm were averaged over at least three repeated measurements.

### Thermal transition midpoints (*T*_m_) from intrinsic fluorescence

The *T*_m_-values of TK variants were measured in an UNcle (Unchained Laboratories, Wetherby, UK) via their intrinsic fluorescence (mainly from tryptophans), expressed as the ratio of fluorescence at 330 nm over 350 nm. The microcuvette arrays were loaded with 9 µL of 1.0 mg/mL TK sample in 50 mM Tris-HCl, 2.4 mM ThDP, 9 mM MgCl_2_, pH 7.0, and excited with a 266 nm laser. The fluorescence ratio was measured as a function of temperature in the range of 30–90 °C with steps of 1 °C, equilibration time of 30 s at each temperature, and a temperature tolerance of 0.5 °C. In most cases, the fluorescence data at the very lowest and highest temperatures were removed to enable fits to use only the most linear portions of the baselines above and below the transition, using.

$${{\text{I}}_{\text{T}}} = \left( {\frac{{\left( {{{\text{I}}_{\text{N}}} + {\text{aT}}} \right) + \left( {{{\text{I}}_{\text{D}}} + {\text{bT}}} \right){\text{K}}}}{{1 + {\text{K}}}}} \right)$$

and

$${\text{K}} = \exp \left[ {\frac{{\Delta {{\text{H}}_{vh}}}}{{\text{R}}}\left( {\frac{1}{{{{\text{T}}_{\text{m}}}}} - \frac{1}{{\text{T}}}} \right)} \right]$$

where *K* is the equilibrium constant for the transition between the native and denatured state; *T* is the experimental temperature; *I*_*T*_, *I*_*N*_ and *I*_*D*_ are the spectroscopic signals of the protein at each T, and for the fully native and fully denatured states, respectively. *a* and *b* are the baseline slopes of the native and denatured region of the curve. *T*_*m*_ is the temperature at which the protein is half denatured; *ΔH*_*vh*_ is the van’t Hoff enthalpy and *R* is the gas constant. All temperature terms in this equation are absolute temperatures in *Kelvin*.

### MD simulation

Molecular dynamics simulation software Gromacs 2020.4 was used to investigate the structural flexibility of wild-type TK and variants. The crystal structure of WT TK (1QGD) was mutated at position 316 to tfm-Phe and 4 F-Phe to generate TK-tfm-Phe and TK-4 F-Phe, respectively, using the Swiss-Sidechain-plugin (SIB Swiss Institute of Bioinformatics) in Pymol (Schrödinger, USA). Swiss-Sidechain-plugin in Gromacs was applied to generate topologies and parameters to run MD simulations using the CHARMM force-field^31^. The starting structure for MD was solvated in a cubic simulation box with water, then neutralised by sufficient Na^+^. The entire system was energy minimised using the steepest descent method (2000 steps) and the conjugate gradient method (5000 steps). Two-phase equilibration was carried out under 50 ns-NVT and 50 ns-NPT ensembles. Finally, a 100 ns MD simulation was performed as six replicates on the whole system at 300 K, where each replicate was independently seeded at random. RMSD was calculated for the backbone using the initial structure as a reference. RMSF of the whole protein was calculated using the last 60 ns of the trajectory and used to analyse the local flexibility of the protein.

### Analysis of DCCM

Dynamics cross correlation matrices were computed using Bio3D^32,33^. The last-10 ns trajectory from each MD simulation was saved at every 10 ps and converted to a dcd file type with the VMD plugin CatDCD^34^. The dcd file was the input for Bio3D and the Cα atom of the protein was selected for calculating the correlation coefficient. Dynamics correlation matrices were averaged from triplicate trajectories, and visualised using Origin.

## Results and discussion

### Expression of TK incorporating fluorinated nnAAs

**Fig. 1:**
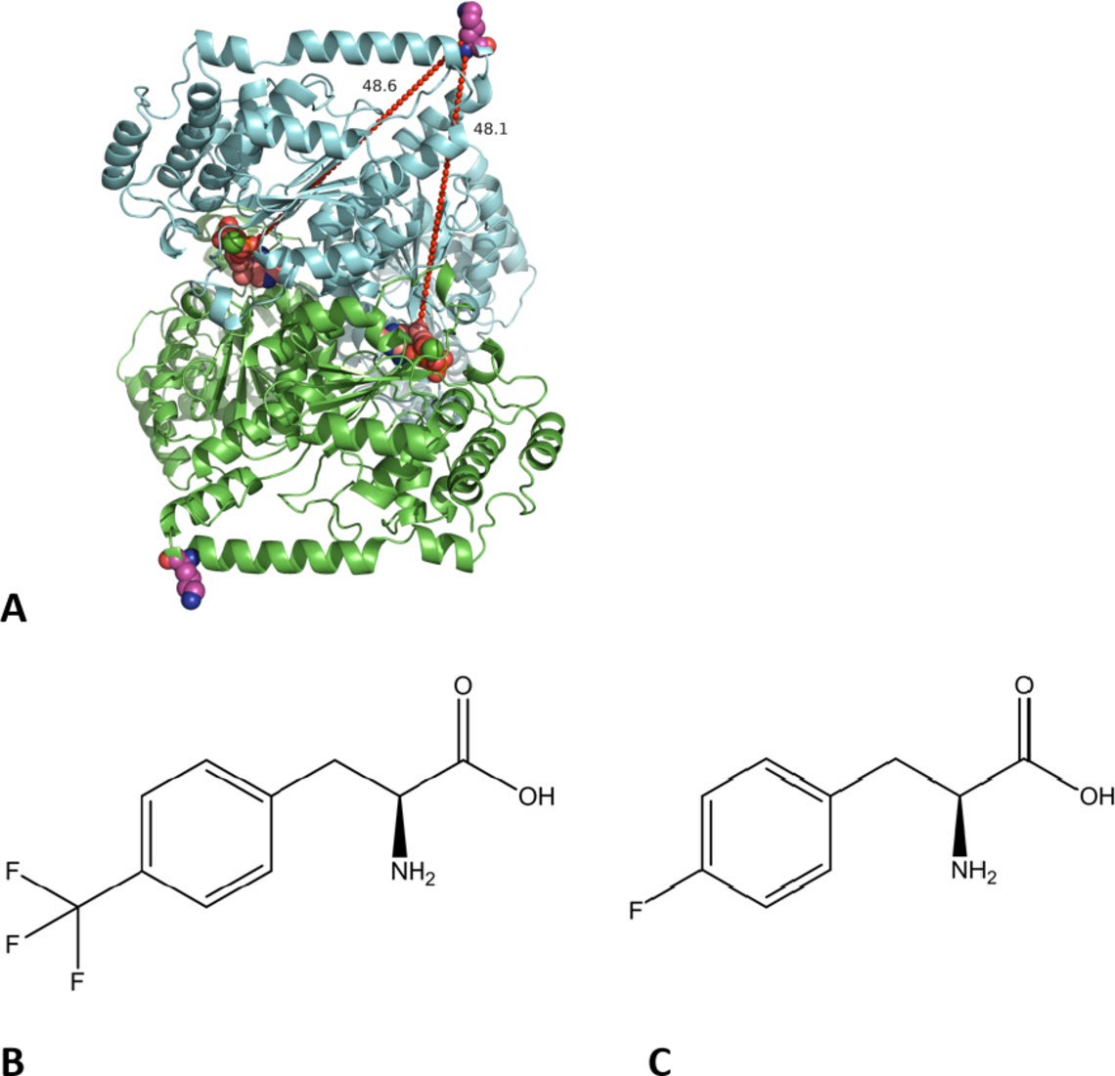
Transketolase structure and the location of residue K316 used for ^19^F probe incorporation. (**A**) Transketolase homodimer, showing ThDP cofactors as salmon spheres, Mg^2+^ as green spheres and the K316 residue as pink spheres. The distances from the K316 C_α_ atom to each ThDP are shown in Å. The two ^19^F probes were incorporated as non natural amino acids (**B**) 4-trifluoromethyl-L-phenylalanine (tfm-Phe), and (**C**) 4-fluoro-L-phenylalanine (4 F-Phe).

Our original aim was to incorporate a ^19^F-labelled amino acids into transketolase (TK) for the study of stability and aggregation, extended to within complex cellular milieu, and so this was desired to be located at a solvent exposed site as far as possible from the enzyme active site. This aimed to minimise the impact of the label on structure and activity. Residue K316 was identified as a site for incorporation of the ^19^F label, at a highly solvent exposed “apex” of the protein on the C-terminal end of the final α-helix in the PP domain (Fig. 1A). Thus it was positioned at the sequence boundary of the PP and Pyr domains, and was equidistant (48.1 Å and 48.6 Å) from the two bound ThDP cofactors, making it one of the furthest possible residues from the active site. Only residues A572 or E573 in the C-terminal domain are potentially further from both cofactors (47.9 Å and 57 Å), but their sidechains were not very solvent exposed, and so were considered unlikely to have been non-disruptive to enzyme structure and stability, and also unlikely to allow for good solvent-exposed side-chain flexibility for the ^19^F label.

To synthesize a site-directed ^19^F-amino acid labelled TK we chose two ^19^F-containing non-natural amino acids, 4-trifluoromethyl-L-phenylalanine (tfm-Phe), and 4-fluoro-L-phenylalanine (4 F-Phe) (Fig. 1B,C) which each closely resemble the structure of tyrosine and provide strong ^19^F signals. To do this, we followed the well established Furter system that allowed us to site-specifically incorporate tfm-Phe and 4 F-Phe nnAAs into the TK gene^35^. While choosing nnAAs, it is important that they are sufficiently different in structure from the natural amino acids so that they are not recognized by normal *E. coli* synthetases, and so the nnAA incorporation will be not only site specific but also site directed. An amber stop codon (TAG) was introduced at the codon encoding for K316, by site directed mutagenesis of the TK gene within the pQR791 plasmid, and confirmed by nucleotide sequencing. Co-transformation of the mutated pQR791 with the pULTRA plasmid into the C321.ΔA.exp amberless *E. coli* strain, followed by overexpression of TK in the presence of tfm-Phe or 4 F-Phe, then His_6_-tag IMAC purification, gave a good yield of high purity ^19^F labelled proteins. The modified TK protein was confirmed by ESI-MS to have the correctly increased masses.

A degree of mis-incorporation of phenylalanine, tyrosine and even glutamine has been known to occur at the site intended for the nnAA^10^. Intact LC-MS for TK-4 F-Phe indicated almost 100% incorporation of 4 F-Phe, which existed in a range of Cys/Met-oxidized forms, and also approximately 20% in the fMet form of TK-4 F-Phe (Figure S1A, Supporting information). LC-MS for TK-tfm-Phe indicated 84% incorporation of tfm-Phe, in a similar range of Cys/Met-oxidized forms, and with 6% as the fMet form of TK-tfm-Phe (Figure S1B, Supporting information). TK-tfm-Phe contained 9% as K316F and 7% as K316Y misincorporation. Neither sample contained any measurable amount of the WT TK. The level of oxidised forms was similar between the two samples and also to previous analyses of WT TK^10^.

### Initial velocities of TK enzyme variants at various temperatures

**Fig. 2:**
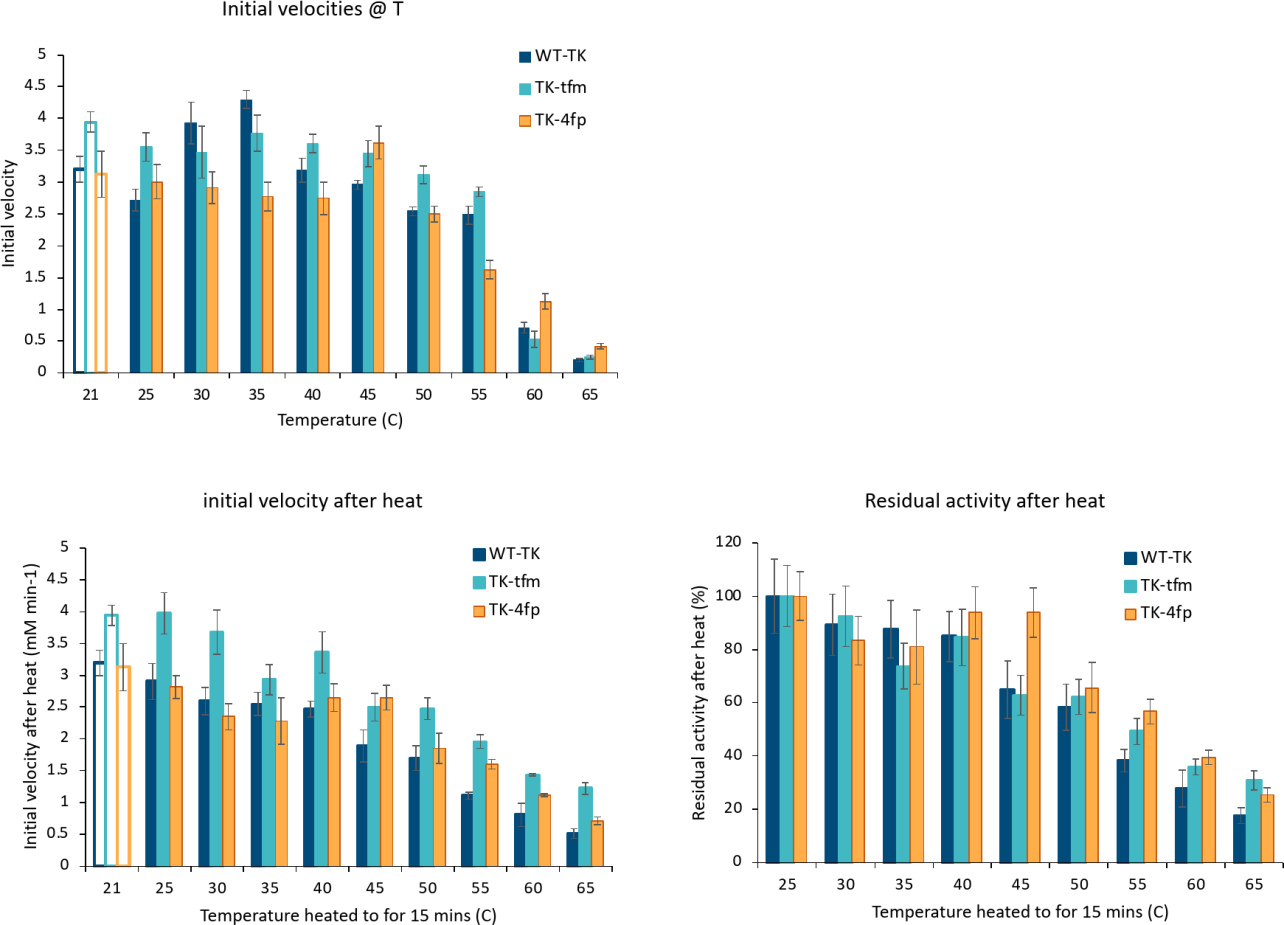
Initial velocities of enzyme variants for reactions (**a**) at various temperatures, and (**b**) at 21 °C but after 15 min heat shocks at various indicated temperatures. (**c**) Residual activities expressed as the initial velocity obtained after heating at each temperature relative to that obtained at 25 °C (in percent). Open bars in (**a**) and (**b**) are values calculated at 50 mM from enzyme kinetics data obtained at 21 °C with the same experimental set up.

The optimum temperature for WT TK activity has been reported in the range of 20–40 °C^10^. The three variants, WT TK, TK-tfm-Phe, and TK-4 F-Phe were tested for activity at 0.1 mg/ml, with 1.36 mM ThDP, 5.1 mM MgCl_2_, 33 mM Tris.HCl, pH 7.0 and 50 mM each of glycolaldehyde and HPA substrates, at temperatures ranging from 25 °C to 65 °C, to determine the effect of temperature on initial velocity, based on the formation of erythrulose measured by HPLC. As shown in Fig. 2a, the initial velocities for TK-tfm-Phe were 15–30% higher than for WT TK under most conditions. However, at 30 °C and 35 °C the WT-TK activity increased with temperature, whereas those for TK-tfm-Phe and TK-4 F-Phe did not. As the temperature was increased further up to 50 °C, the initial velocity of WT TK declined whereas those for TK-tfm-Phe and TK-4 F-Phe again did not change considerably. Exceptionally, at 45 °C, the initial velocity for TK-4 F-Phe increased by 30%. Finally at 55 °C and above, all initial velocities were found to decrease to almost no activity at 65 °C as expected due to denaturation of the enzyme. Thus, the optimum temperature for WT TK was confirmed at 30–35 °C. By contrast, TK-tfm-Phe retained a much broader optimum from 21 to 45 °C, whereas the optimum temperature for TK-4 F-Phe increased to 45 °C.

### Residual initial velocities of TK enzyme variants at 21 °C, after pre-incubations at various temperatures

To separate the impact of temperature on enzyme catalysis from the effects of irreversible enzyme denaturation, the initial velocities at 21 °C were also determined *after* a pre-incubation for 15 min at various temperatures. We used 21 °C as 21–25 °C has been typically used in previous literature for TK enzyme kinetics. As shown in Fig. 2b, the initial velocities after heating at 21–45 °C were again 15–42% higher for TK-tfm-Phe than for WT-TK. By contrast, those for TK-4 F-Phe remained similar to WT-TK under all conditions except at 45 °C where the activity of TK-4 F-Phe remained 40% higher. This increase at 45 °C for TK-4 F-Phe provided an independent confirmation of the related increase observed for the initial velocity at 45 °C in Fig. 2a.

Figure 2c presents the same data as the % activity retained after heating compared to the initial velocity at 25 °C. All three enzymes retained only 17–40% of their initial activity, when pre-incubated at 60 °C and 65 °C, consistent with previous work in which WT TK was shown to denature with a thermal transition midpoint at 58.3 °C at pH 7.5, and kinetically with a half-life of 3.8 min at 65 °C and 30 min at 60 °C^1^. Thus it was clear that increasing the temperature from 25 to 45 °C did not significantly increase the enzyme kinetics, except for WT TK at 30 to 35 °C, and for TK-4 F-Phe at 45 °C. There was a slight decline in active enzyme for TK-tfm-Phe and WT TK at least, as the temperature increased to 45 °C. However, enzyme denaturation only became significant at 50 °C and above on this experimental timescale. For WT TK the increased enzyme kinetics at 30 to 35 °C seemed to be driven by temperature-dependent reaction kinetics alone and not through any structural changes, given that the initial velocity determined directly at those temperatures increased, but residual activity after heating did not. For TK-4 F-Phe, the increase in initial velocity at 45 °C was not part of a general trend in reaction kinetics, and appeared to be driven through a protein stabilising event as evidenced by the increased residual activity.

Heat activation events have been observed previously with WT TK at 0.5 mg/ml and pH 7.5, but only at low (unsaturating) cofactor concentrations (0.5 mM MgCl_2_ and 0.05 mM ThDP), where the catalytic activity of the enzyme increased almost three-fold through a heat activation step prior to carrying out the enzyme reactions^1^. Further investigations had found this heat-activation to be greatest at low ThDP concentrations and resulted from a heat-induced shift towards a conformation of TK with a higher affinity for ThDP, shifting the equilibrium towards holo-TK and an observed increase in activity^22^. By contrast, the improvement of activity was no longer apparent at 2.4 mM ThDP because this was sufficient to saturate even the low affinity conformation^22^. For the current work at 1.36 mM ThDP, 5.1 mM MgCl_2_, the ThDP cofactor would already have reached > 90% saturation of the low-affinity form of the WT-TK enzyme (*K*_d_ ≈ 400 µM at 5 mM MgCl_2_), which itself accounted for 70% of the total enzyme based on the previous data. Thus, heat-activation in these conditions could only at most improve the fraction of fully active enzyme by 7%, which is consistent with the lack of heat activation for WT-TK in Fig. 2b. By contrast, TK-4 F-Phe showed a clear heat-activation at 40–45 °C, suggesting either that the ThDP *K*_d_ for the low-affinity form of this variant had increased, or that the proportion of enzyme starting in the low-affinity form had increased.

The influence of non-natural amino acid mutations in close proximity to the active site have been demonstrated previously, resulting in up to forty-fold increases in activity compared to wild types^10,22,36^. By contrast, mutations that influence activity far from the active site are less common^19,37^, though they do occur for example in directed evolution experiments, and are understood to exert their influence through strongly correlated dynamics mediated via structural networks^3^. However, the observation that ^19^F-labelling at a highly solvent-exposed residue that was 47 Å from either active site could enhance enzyme activity up to 42% and modify the optimum temperature profile, was still a surprising result.

### Enzyme kinetic parameters of TK-tfm-phe and TK-4 F-Phe

To understand whether the fluorinated phenylalanine nnAAs were altering enzyme catalysis through turnover or substrate binding, and to explore stability effects, we also determined enzyme kinetic parameters before and after a thermal heat challenge, for both variants and WT TK, using a constant concentration of HPA but varying GA. The kinetic parameters from Michaelis-Menten fitting are shown in Table 1.

The 15 min heat step at 50 °C was found to have no significant impact on the kinetic parameters obtained for the WT or variants, except for a 20±14% increase in *k*_cat_ and 39±18% decrease in *K*_m_ for TK-4 F-Phe which provided a 35±14% increase in the initial velocity at 50 mM GA. This was consistent with the increased initial velocity observed both directly at 45 °C (Fig. 2a) and after heating for 15 min at 45 °C (Fig. 2b) as measured independently for TK-4 F-Phe. Such an increase in *k*_cat_ would be compatible with the known influence of heating on the affinity for ThDP in the low-affinity form of the enzyme. As described above, this potential to be heat-activated when using 1.36 mM ThDP, 5.1 mM MgCl_2_ would be consistent with an increase in the ThDP *K*_d_ for the low-affinity form of this variant, or an increase in the proportion of enzyme starting in the low-affinity form.

**Table 1:** Enzyme kinetic parameters for TK WT and variants. Enzyme kinetics were determined at 50 mM HPA, 0.1 mg/ml TK, 1.36 mM ThDP, 5.1 mM MgCl_2_, and 33 mM Tris.HCl, pH 7.0 based on 3–6 sample repeats. ^a^v_0_ calculated at 50 mM GA directly from the kinetic parameters. Standard errors are shown in parentheses.

	K_m_ (GA) (mM)	V_max_ (mM min^− 1^)	k_cat_ (min^− 1^)	k_cat_/K_m_ (mM^− 1^ min^− 1^)	v_0_ calculated at 50 mM (mM min^− 1^)^a^
WT TK 21 °C	22 (3)	4.6 (0.2)	3200 (150)	150 (20)	3.2 (0.2)
WT TK 50 °C	18 (2)	4.8 (0.2)	3320 (120)	180 (21)	3.5 (0.2)
TK-tfm-Phe 21 °C	14 (1.5)	5.1 (0.1)	3530 (110)	250 (30)	3.9 (0.1)
TK-tfm-Phe 50 °C	14 (2)	4.3 (0.3)	2980 (200)	200 (35)	3.3 (0.3)
TK-4 F-Phe 21 °C	18 (3)	4.3 (0.4)	2980 (320)	160 (30)	3.1 (0.3)
TK-4 F-Phe 50 °C	11 (1)	5.1 (0.4)	3580 (300)	320 (40)	4.2 (0.3)

The impact of the two nnAAs on kinetic parameters relative to WT TK were very different. TK-4 F-Phe did not significantly affect the enzyme kinetics at 21 °C. By contrast, for TK-tfm-Phe, the *K*_m_ decreased by 36±16% resulting in the 22±7% higher initial velocity at 50 mM GA relative to WT TK at 21 °C, and an associated increase in *k*_cat_/*K*_m_. These data were consistent the initial velocity measurements made independently for different temperatures in Fig. 2, in particular for TK-tfm-Phe which had 15–30% higher initial velocities across the temperature range of 25–55 °C. It also clarified that the initial velocities of TK-4 F-Phe and WT TK were more similar in the absence of heat activation.

### Effects of ^19^F-nnAAs on enzyme stability

To confirm whether or not the ^19^F-labelling with phenylalanine analogues directly affects conformational stability, we determined the thermal transition midpoint temperatures (*T*_m_) of the variants and WT TK from thermal scanning fluorimetry (Figure S2, Supporting information) over the temperature range 20–90 °C at 0.1 mg/ml TK, in 50 mM Tris-HCl, 2.4 mM ThDP, 9 mM MgCl_2_, pH 7.0. The WT TK had a *T*_m_ of 56.7 °C consistent with previous measurements of 58.3 °C in a slightly different buffer (25 mM Tris–HCl, pH 7.5, 0.5 mM MgCl_2_, 0.05 mM ThDP)^1^. By comparison, TK-tfm-Phe and TK-4 F-Phe had *T*_m_ values of 64.1 °C and 60.8 °C, respectively, which were 4–7 °C higher than for WT TK (Table 2). Therefore, the incorporation of ^19^F-nnAAs at the position of residue K316 increased the thermal stability regardless of the specific nnAA used, but this was much greater for TK-tfm-Phe. These differences in stability did not correlate with changes in enzyme kinetics (*k*_cat_, *V*_max_, *K*_m_ or initial velocities), and so it is unlikely that those changes were caused by differences in partial enzyme denaturation or aggregation.

**Table 2:** Stability of the WT-TK and variants as determined by their thermal midpoint transitions (*T*_m_) and proportion of native enzyme remaining after heat treatments at 4 °C, 21 °C and 50 °C. Native proportion includes homodimeric and monomer enzyme as measured by SEC. These were combined because dilution during SEC can cause partial dissociation to monomer. Soluble aggregates were determined from non-native peaks. Peak area lost was obtained by subtracting the total peak area from the total at 4 °C. Standard errors are shown in parentheses.

	DSF	SEC								
		Native (%)			Soluble aggregate (%)			Peak area lost (%)		
	T_m_ (°C)	4 °C	21 °C	50 °C	4 °C	21 °C	50 °C	4 °C	21 °C	50 °C
WT	56.7 (0.07)	97.2	97.3	9.3	2.8	2.7	28	0	0	63
TK-tfm-Phe	64.1 (0.1)	100	81.9	61.5	0	4.9	1.7	0	13	36.8
TK-4 F-Phe	60.8 (0.1)	99.9	99.4	90.7	0.1	0.6	9.2	0	0	0

Variants were also analysed by Size Exclusion Chromatography (SEC) at 21 °C, after taking them out of storage at 4 °C, heating for 15 min at 21 °C and 50 °C, and then compared to the original samples stored at 4 °C (Figure S3, Supporting information). The proportion of native enzyme remaining in each sample is shown in (Table 2) along with the soluble aggregates and total loss of peak area due to large aggregates that did not even enter the column. For WT TK treated at 21 °C the sample loss was negligible, but when pre-treated at 50 °C and re-cooled, there was a significant loss of approx. 91% of the native enzyme, most of which formed aggregates too large to appear in the chromatograms. The presence of larger aggregates was also confirmed by DLS for the WT-TK sample pre-treated at 50 °C (Figure S4, Supporting information).

TK-tfm-Phe was less stable against monomer loss at 21 °C, retaining 81.9% native enzyme, compared to the same protein kept at 4 °C. However, heating at 50 °C led to only 38.5% native enzyme loss, primarily through the formation of aggregates. TK-4 F-Phe was more stable overall at 21 °C and 50 °C, with 99.4% and 90.7% native enzyme remaining respectively, and none of the enzyme lost to large aggregates in any condition.

Therefore, SEC confirmed that at 21 °C, the variants were similar to WT TK with a small amount of aggregation. However, at 50 °C WT TK was more prone to deactivation by aggregation than the other two variants, and TK-4 F-Phe was the most stable to 50 °C heat treatment. These data were consistent with the residual activities after heating (Fig. 2c) which did not decrease for TK-4 F-Phe until 50 °C, and were always higher than for the other variants. The loss of 91% of WT TK as measured by SEC was more dramatic than the 40% loss in initial velocity (60% residual activity) (Fig. 2c), and the unchanged kinetic parameters (Table 1), but consistent with the lower *T*_m_ (56.7 °C). These differences are likely due to having used a heat challenge of 50 °C which is very close to the *T*_m_ for WT, which makes the experiments for WT TK in particular, very sensitive to the precise time taken for the samples to equilibrate to 50 °C during the 15 min incubations. Additionally, it is possible that the 63% and 36.8% peak area lost (to larger aggregates not entering the SEC column) for WT TK and TK-tfm-Phe respectively, is related to their 42% and 38% losses in initial velocity after heating at 50 °C, implying that the soluble aggregates remained active. Either way, it is clear that the nnAAs had positive impacts on both conformational stability and aggregation behaviour compared to WT TK.

One other factor to consider is the purity of the variants. While TK-4 F-Phe did not contain any mis-incorporated amino acids at residue 316, the TK-tfm-Phe variant contained some K316F and K316Y which accounted for 16% of the enzyme population. It cannot be ruled out that this population contributed to stability and aggregation differently to the 84% TK-tfm-Phe population, including the possibility that they also seed aggregation of the TK-tfm-Phe population. Potentially this may have accounted for the lower native fraction for this variant (81.9%) at 21 °C, as measured by SEC. However, we did not investigate this further.

### ^19^F and ^1^H NMR studies

While the original intention was to use an ^19^F labelling strategy and ^19^F-NMR to probe aspects of TK stability under different conditions, it was now interesting to probe the observed differences in temperature-dependent activity and/or stability induced by the ^19^F-labelling mutations themselves. The presence of fluorine in TK-tfm-Phe and TK-4 F-Phe, and hydrogen atoms in these variants as well as in WT, allows convenient use of both ^19^F and ^1^H NMR spectroscopy for observing temperature-dependent spectral changes that report on changes in protein structure and dynamics.

**Fig. 3:**
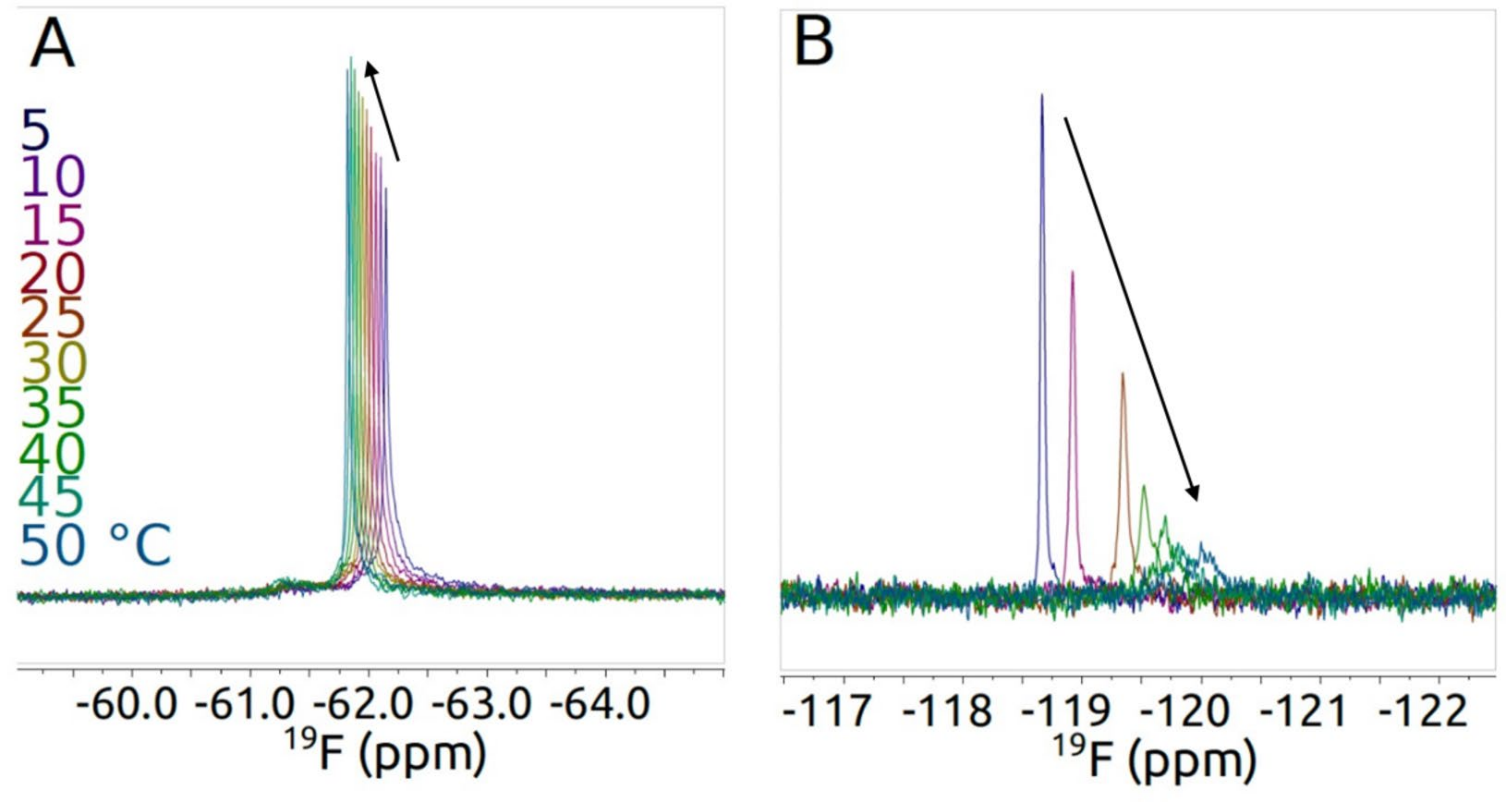
^19^F-NMR spectra of TK variants at a series of indicated temperatures.^19^F-NMR spectra were recorded at 15 mg/ml TK-tfm-Phe (**A**) or TK-4 F-Phe (**B**) in 2.4 mM ThDP and 9 mM Mg_2_^+^, 20 mM Tris.HCl, pH 7.0, containing 10% D_2_O and 0.01% TSP. All 1D^1^H and^19^F –NMR spectra were recorded for 0.22 mM protein solutions on 500 MHz spectrometer, with spectra referenced based on TSP calibration of^1^H spectra. Processed with 5 Hz exponential line-broadening. Arrows denote the direction of change in the peak position with increasing temperature.

**Fig. 4:**
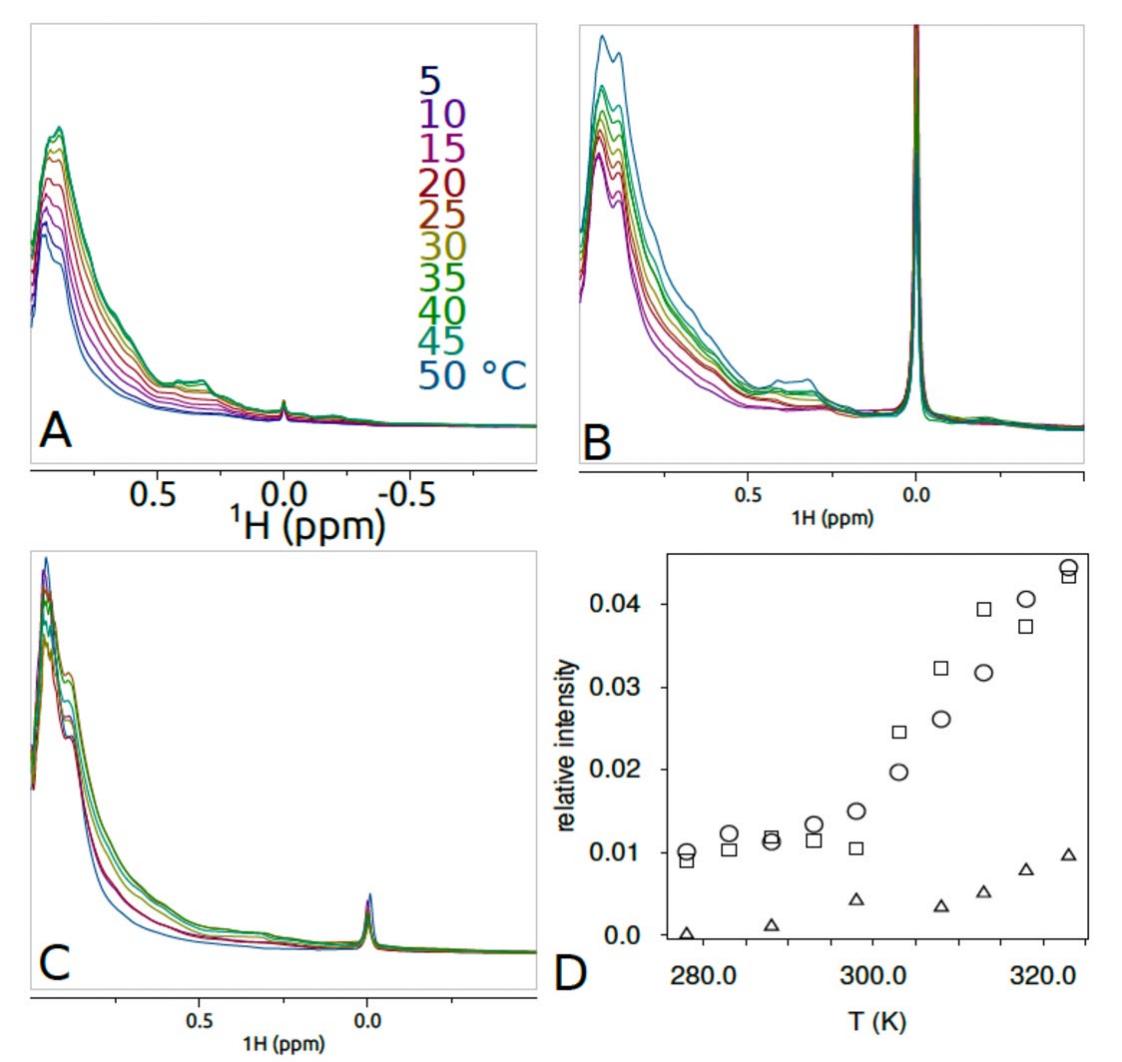
^1^H-NMR: “Methyl” region of^1^H NMR spectra of TK variants displayed between 1 and − 1 ppm at the indicated temperatures^1^. H NMR spectra were recorded at 15 mg/ml of (**A**) WT TK, (**B**) TK-tfm-Phe (**B**) or (**C**) TK-4 F-Phe in 2.4 mM ThDP and 9 mM Mg_2_^+^, 20 mM Tris.HCl, pH 7.0, containing 10% D_2_O and 0.01–0.2% TSP, recorded at 500 MHz. Water suppression with excitation sculpting, 128 scans, spectral width 16ppm, acquisition time 2 s, offset 4.7 ppm. Spectra referenced to TSP. Processed with 5 Hz exponential linebroadening. (**D**) Temperature dependence of the peak intensity ratio for (ϒ) WT, (Δ) TK-4 F-Phe, and (ο) TK-tfm-Phe, each obtained from the peak integrals (using automated baseline correction) of the small peaks at 0.42 and 0.3 ppm relative to the large peak at 0.9 ppm arising from non-dispersed methyl groups.

The observed ^19^F spectra confirmed successful incorporation of fluorine into both variants, giving an ability to monitor these resolved signals without the complexity of signal overlap, or interference from background signals. For each construct, spectra were recorded at a number of temperatures (see Fig. 3). It should be noted that ^19^F spectra will report on the mobility change of the fluorine tag itself at the mutation site as well as any effects of the local surroundings. For a large protein such as TK, the ^1^H spectrum presents an envelope of multiple hydrogen signals from the whole molecule, and is mostly affected by the factors such as overall molecular motion and the state of molecular association. Signals from individual sites are not resolved in this case, however several methyl signals characteristic of folded 3D structure could be identified at around 0.3-0.4ppm (Fig. 4) which could be used as reporters for temperature-related changes. Overall, the fluorine modifications and aromatic ring of the phenylalanine analogues did not visibly perturb the folding of TK.

As the temperature was increased from 5 °C to 50 °C, the ^1^H reporter signals in all three constructs increased and sharpened due to faster molecular tumbling and increased molecular motions (Fig. 4A-C). In order to compensate for these effects, the sum of ^1^H peak integrals for the dispersed methyl groups (~ 0.15–0.55 ppm) were normalised to that of the large peak for non-dispersed methyls (0.55-1.05ppm), (Fig. 4D) to give a peak intensity ratio. The signal-to-noise for the “dispersed” 1 H peak integrals at 50 °C was 158:1, 52:1, and 110:1 for WT TK, TK-4 F-Phe and TK-tfm-Phe, respectively, as determined by integrating the baseline over a similar frequency range, and using Bruker .sino macro. These peaks should be equally affected by the viscosity and direct molecular motion effects, and so the ratio, yielding relative intensity of representative methyl signals, is dependent on the population of the conformational species and local dynamics. The relative intensity increased as the temperature increased, indicating a greater population of species with faster local dynamics. This could indicate a transition from a higher order oligomeric state or from an ensemble state such as a “molten globule” with rapidly interconverting structure, to a better folded, monomeric state. The transition to this state did not appear to be complete before temperature denaturation began to dominate at above 50 °C. The TK-tmf-Phe variant behaved broadly the same as WT TK with the largest intensity changes that started at above 300 K (27 °C). The TK-4 F-Phe variant did not make this transition to the same extent (triangles in D), and also started at a higher temperature of > 310 K (37 °C). Therefore, ^1^H-NMR data suggests quite a different dynamic response of the enzyme variants to increased temperature, hinting at the presence of different underlying molecular motions^37^.

These data suggest a “re-structuring” of some type at > 300 K, and this would be consistent with the known heat activation events measured previously at > 40 °C for WT^1,21^. It should be noted that heat activation of WT or TK-tfm-Phe was not observed in the current study because the cofactor concentration was too high to observe it^22^, but the related structural changes would still be expected to occur. The smaller changes and at the higher temperature of > 310 K (40 °C) for TK-4 F-Phe, are also consistent with the observed slight heat activation at 40–45 °C for that variant.

The ^1^H signal intensity changes described above may be the result of not only intramolecular structural changes. Indeed, given that heating to 50 °C also leads to aggregation in WT, to a lesser extent in TK-tfm-Phe, and much less so in TK-4 F-Phe, then some of the ^1^H signal intensity change may relate to the formation of aggregation-prone conformations of TK. The smaller signal intensity change for TK-4 F-Phe and the relative stability to aggregation for this variant would be consistent with this hypothesis.

The analysis of ^19^F signals (Fig. 3) from the mutation site reveal complementary information and again show differences in dynamic response of TK-tfm-Phe and TK-4 F-Phe to increased temperature. First, the temperature gradients of ^19^F signals in these two mutants had opposite signs, with 4 F-Phe shifting − 100 ppb/K, and tfm-Phe shifting + 10 ppb/K. This suggested not only differences in the immediate chemical environment, but also differences in how the immediate chemical environment reacted to the temperature increase. Moreover, whereas the ^19^F signal in TK-tfm-Phe remained sharp, largely retaining its intensity as temperature was raised, the signal from TK-F-Phe got significantly broader, losing its intensity at higher temperature. This shows that at higher temperature the 4 F-Phe at the mutation site gained additional intermediate timescale dynamics and perhaps lost any uniquely defined structure around the mutated residue. For TK-tfm-Phe at higher temperature a new broad ^19^F signal appears downfield to the main signal, suggesting existence of multiple structural forms which could include the aggregates observed at 50 °C. It is also possible that the chemical and steric differences between the tfm-Phe and 4 F-Phe sidechains are responsible for the differences in these behaviours, although this cannot be deconvoluted from potential changes in the dynamics of the structure in the local environment. Finally, it is also possible that hydrogen bonding to fluorine may have contributed to the observed shifts in ^19^F signals, although such bonding has only been observed previoulsy on rare occasions such as for *ortho*-fluorinated phenylalanine derivatives at low temperatures of 90 K^38^, or for fluoro-organic compounds in solid state^39^. However in the current study the contribution of such bonds to stability is unclear.

### Molecular dynamics simulations

The improved *v*_0_ and *K*_m_ of TK-tfm-Phe over WT-TK, and the stronger heat-activation effect of TK-4 F-Phe at 45 ^o^C, suggests some long-range impacts of the mutations at K316 on the enzyme active-site. To probe the impact of these mutations on native enzyme dynamics, we carried out molecular dynamics simulations at 300 K over 100 ns. In all simulations, the system was determined to have equilibrated fully within the first 40 ns when the RMSD reached a plateau (Figure S5, Supporting information). Root mean square fluctuation (RMSF) was thus calculated from the simulation frames from 40 to 100 ns.

**Fig. 5:**
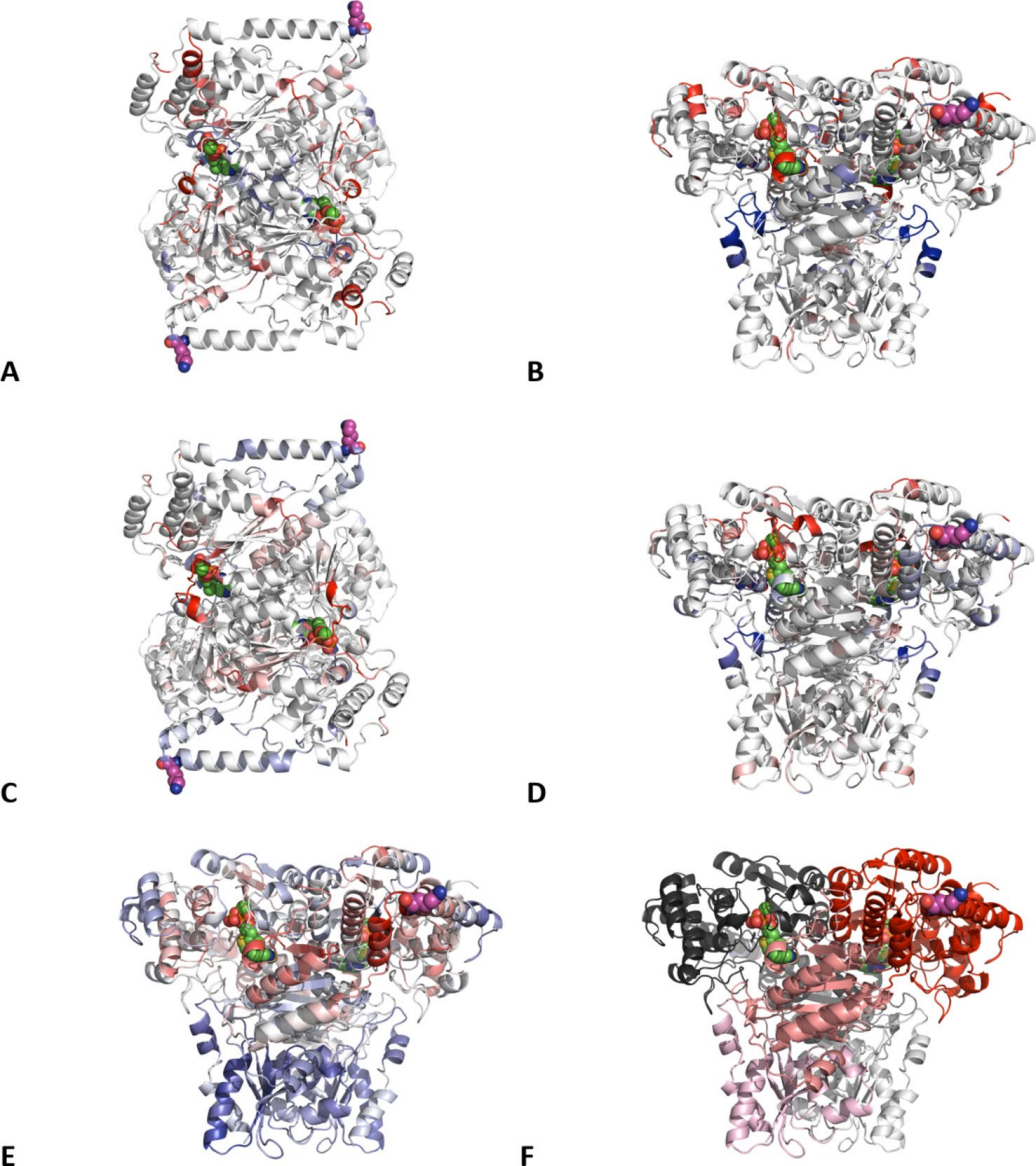
Significant changes in residue-level RMSF due to mutations, and correlation of dynamics to residue K316 dynamics obtained from MD simulations. K316 mutation site is shown as magenta spheres. Cofactors TDP and Ca^2+^ are highlighted as green spheres. Changes in RMSF (ΔRMSF) at each residue for (**A**,**B**) TK-tfm-Phe, and (**C**,**D**) TK-4 F-Phe are calculated as RMSF_var_ - RMSF_wt_ and with significant values shown from 0.03 (red), through 0.0 (white) to -0.03 (blue). Values where ΔRMSF < σ_m_ the standard error of mean are set to 0.0 and coloured white. A and B show top and side views respectively, as do C and D. (**E**) Dynamical cross-correlation matrix (DCCM) coefficients coupled to residue 316, coloured from blue (negative) through white (0) to red (positive). Data were generated from 12 replicas for each variant using 6 simulation repeats and combining both monomers from the homodimer. (**F**) TK coloured by chains (grey vs. coloured) and domains (PP - dark, Pyr - mid, C-term - light).

### Residue-level changes in RMSF

Decreased RMSF indicates less mobility and so increased local stability. The average RMSF across all residues for each of the three variants did not correlate particularly well with their *T*_m_, *K*_m_ or *V*_max_ values, although TK-tfm-Phe had the highest *T*_m_ and lowest RMSF. However, there was a good linear correlation (R^2^ > 0.8, *n* = 3) between RMSF and *V*_max_ for a third of the residues. Most of these were in the C-terminal domain, with a few in localised specific structural regions of the PP and Pyr domains, notably including a short helix in the active site (L382-L387) for which the RMSF increased with *V*_max_. It will be interesting to see in future whether such correlations persist for a wider range of variants. For the current study it was at least useful to identify which regions of structure had altered RMSF as a result of the mutations.

For TK-tfm-Phe, the mutation decreased the RMSF for K316 and adjacent residues as shown by the blue region in Fig. 5A. Further from the mutation site, the rest of the PP domain showed a few particular regions with small increases in RMSF. These were at the N-terminus, but also cofactor-binding loop 1 (S188-H192), and the short active site helix (L382-L387). Some particular regions also showed strong local decreases in RMSF, notably in a buried active site forming loop (D462-P475), and a structurally adjacent loop and helix in the C-terminal domain region (G636-E647). Mutations at some of these sites, including D469E, D469T, S188Q were previously found to improve enzyme activity^14^.

For TK-4 F-Phe, the mutation of residue K316 to 4 F-Phe also decreased the RMSF locally, slightly more than it did for TK-tfm-Phe as seen in Fig. 5C and D. The biggest decreases in RMSF were at the buried active site forming loop (H461-D469) and the adjacent C-terminal domain region (G636-E647), as also seen above for TK-tfm-Phe. Compared to TK-tfm-Phe, the TK-4 F-Phe had a more significantly increased RMSF in cofactor-binding loop 1 (S188-H192). The H192P mutation in this region has been previously identified to be highly stabilising^3^.

Thus nearly all major changes in RMSF for both variants were directly involved in catalysis. Any one or more of these could have mediated the observed changes in enzyme kinetics.

### Dynamic cross-correlation matrix (DCCM) analysis

It is now well known that protein dynamics can mediate long-range allostery in proteins and enzymes^40–43^. We have also previously shown how networks of residues within TK can propagate the impacts of mutations over long distances, and that this can be revealed through their correlated motions within molecular dynamics simulations^16^. To quantify and identify the regions dynamically correlated with residue K316, we calculated a Dynamic Cross Corelation Map (DCCM) for all variants. This determined a cross-correlation coefficient (C_ij_) between Cα atoms for all pairs of residues in TK, which then have a value of 1 for highly correlated, -1 for highly anti-correlated and 0 for non-correlated motions. The DCCM maps for all three variants are shown in the supplementary information (Figure S6, Supporting information), and showed little difference between them. This is expected given that while the K316 surface mutations were found to alter the dynamics of residues, they were unlikely to completely disconnect any networks through the removal of key interactions.

Our focus here was only on the dynamics coupled to residue K316 and so we mapped the correlation coefficients paired to this residue, against the WT TK structure in Fig. 2E. It was clear that regions local to the K316 residue in WT TK had highly correlated motions (shown in red) as expected for residues in close structural and sequence proximity. The K316 residue and local dynamics were highly anti-correlated with motions in the whole of the C-terminal domain, and most of the PP domain, which most likely reflects an overall breathing motion of the enzyme in which the solvent-exposed apex of the PP-domain containing residue K316, all periodically pulls away from the rest of the enzyme. This motion directly linked residue K316 to the active site, as it included an extended structural network within the Pyr domain, linking a surface loop (G392-A401), beta strand (P373-S379), and onwards into cofactor-binding loop 2 (A380-K394) which included the active-site residues A380, D381, L382, P384 and S385. The neighbouring cofactor-binding loop1 (Residues 187–198) which is found in the PP domain was found to be strongly anti-correlated to these dynamics, indicating strong coupling but with motions out of phase.

Even if the K316 region and Pyr domain are moving in a concerted way relative to the rest of the enzyme, how do mutations at solvent exposed K316 exert so much influence on local dynamics, that are then propagated to distant dynamics? K316 is highly solvent exposed, and surrounded at the C-terminus of an α-helix, by four alanine residues, which provide very few opportunities for the side-chain of this residue to make local contacts. The only potential for electrostatic interactions come from (correlated) Glu309 two turns away in the same helix (10–11 Å), Glu321 at the N-terminus of the next helix (8–15 Å), or (anti-correlated) His141 which is 8 Å away in a loop that connects K316 to a dynamically correlated PP-domain helix that extends all the way down to the ThDP cofactor. Thus, the mutations to fluorine labelled phenylalanine analogues would each remove these electrostatic interactions if they exist. The addition of the more hydrophobic fluorinated sidechains, and the aromatic ring, would also offer more potential to form Van der Waals contacts with the surface residues in the helices around K316. Several hydrophobic residues, including the four surrounding alanines, have interaction potential such as Y318, P319, and A323, and any of these would decrease the local dynamics as observed by the lower RMSF in both variants. The temperature-dependent changes in ^19^F NMR signals also indicated the formation of alternative local conformers as temperature increased, consistent with restructuring of local interactions with other protein sidechains that were otherwise well-defined at the lower temperatures.

## Conclusions

Two types of ^19^F labels were incorporated as non-natural amino acid analogues at a highly solvent exposed site located at the furthest point from the two identical enzyme active sites. While the overall changes in enzyme kinetics were small compared to those for variants typically obtained by directed evolution, often including mutations far from the active site^19,44^, or for rational mutations made within the active site, they were nonetheless reproducible. A significant impact of the mutations on stability was observed, measured as both the thermal transition temperature (*T*_m_) and as the degree of aggregation at different incubation temperatures, such that the TK-4 F-Phe variant almost abolished aggregate formation at 50 °C, compared to the 63% insoluble aggregates formed for WT-TK. The previously observed heat activation of TK at 40–50 °C was not observed here for WT-TK (or TK-tfm-Phe), although a small activation was observed for TK-4 F-Phe. The lack of activation was as expected due to the high cofactor concentrations used, but any structural events leading to previously measured increases in cofactor affinity, would still be expected to occur. Indeed, ^1^H-NMR indicated structural changes at these temperatures, prior to denaturation or aggregation at higher temperatures, that could be involved in the heat activation events. The relative increase in ^1^H signal intensity for TK-4 F-Phe was smaller, indicating a different overall behaviour and it was hypothesised that this may be related to the improved stability to aggregation for this variant. Finally, molecular dynamics simulations indicated the potential role of correlated protein dynamics through a network of residues that could mediate long-range effects on active site structure, stability and function. This builds on recent studies that have demonstrated such effects in transketolase^2,3,16^.

## Electronic supplementary material

Below is the link to the electronic supplementary material.

Supplementary Material 1

## Data Availability

All data reported here are available on request to the corresponding author.
