# Supplementary material for: Enzyme stabilisation due to incorporation of a fluorinated non-natural amino acid at the protein surface
Source: Sci Rep. 2024 Nov 14;14:28080. doi: 10.1038/s41598-024-79711-6 (PMC11564776; doi:10.1038/s41598-024-79711-6)
Supplement: Supplementary file 1 — Supplementary Material 1 [file 41598_2024_79711_MOESM1_ESM.docx]

**Supplementary Information for:**

**Enzyme stabilisation due to incorporation of a fluorinated non-natural amino acid at the protein surface**

Arka Mukhopadhyay^1^, Yiwen Li^1^, Matthew J. Cliff^2^, Alexander P. Golovanov^3^, Paul A. Dalby^1,*^

^1^Department of Biochemical Engineering, University College London, Gordon Street, London, WC1H 0AH, United Kingdom

^2^Manchester Institute of Biotechnology, 131 Princess St., Manchester, M1 7DN. UK.

^3^ Department of Chemistry, School of Natural Sciences, Faculty of Science and Engineering, The University of Manchester, Manchester M1 7DN, U.K.

**A)**

**
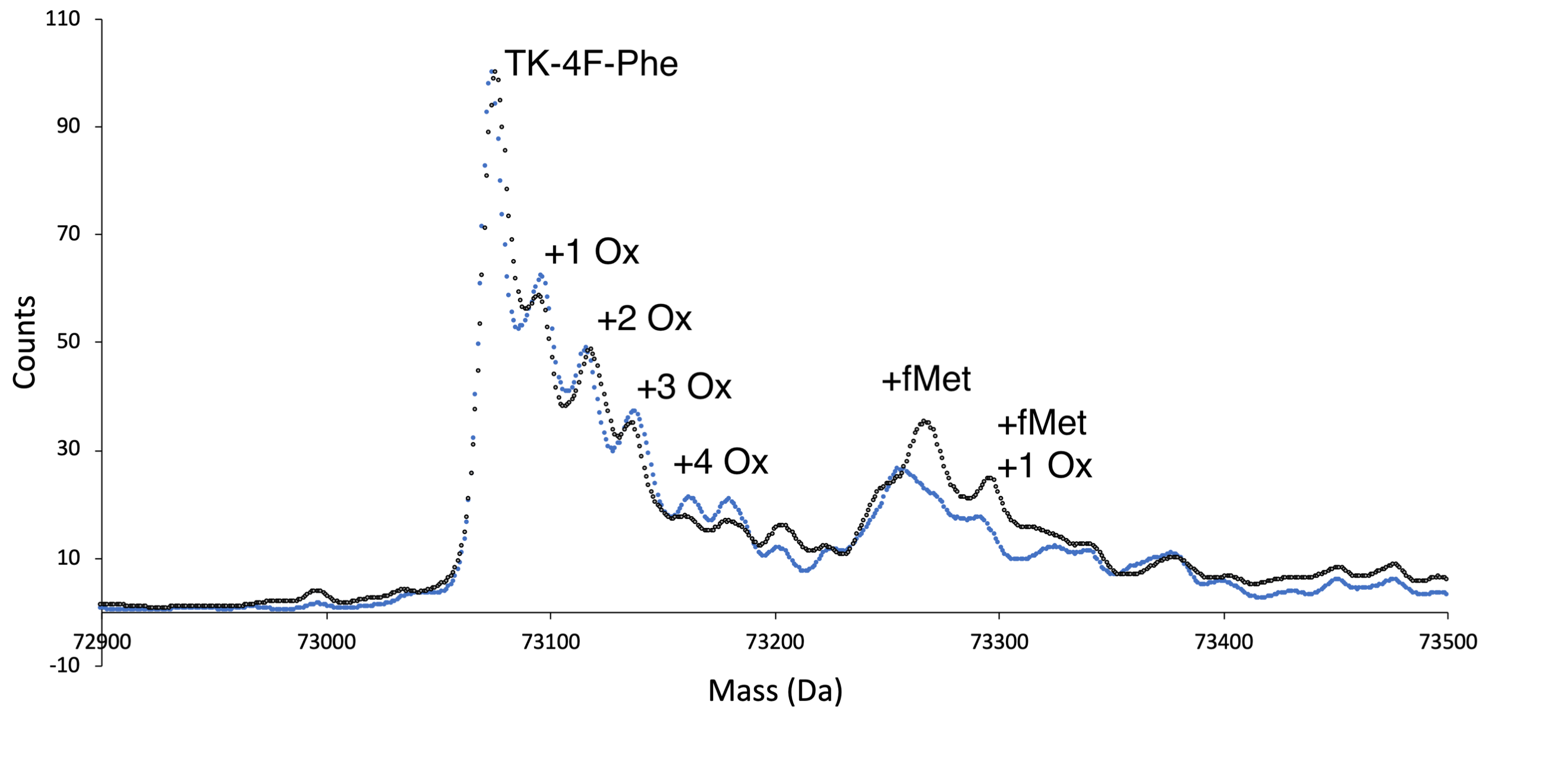
**

**B)**

**
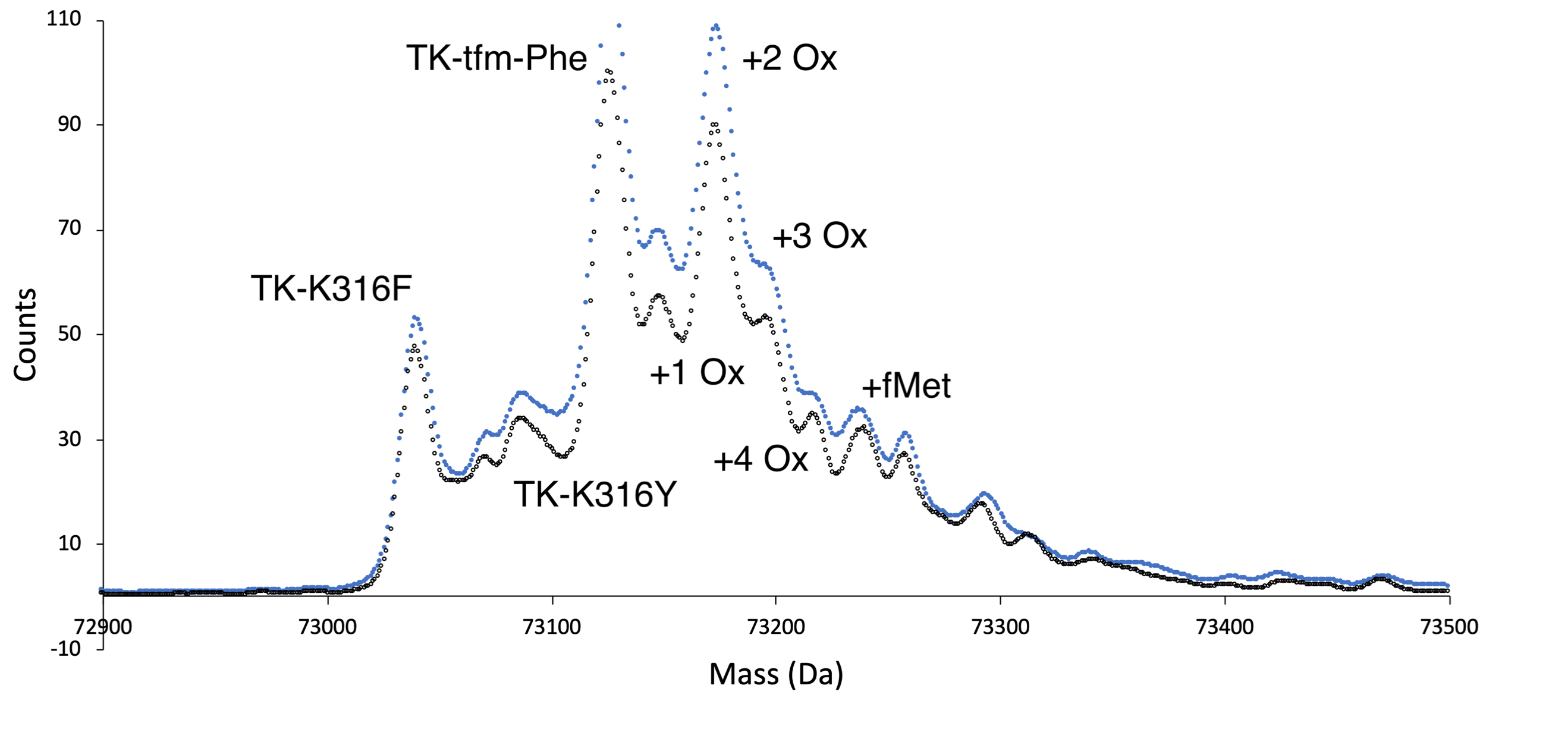
**

**Figure S1. Mass spectra of purified TK variants to identify fraction with correctly incorporated non-natural amino acid.** A) TK-4F-Phe; B) TK-tfm-Phe. Each plot shows two repeat LC-MS sample measurements. Ox = oxidation (+16Da), fMet = formyl-methionine.


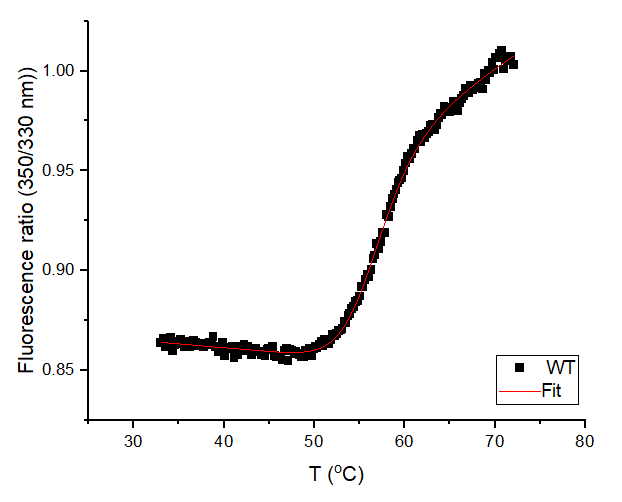
**WT**


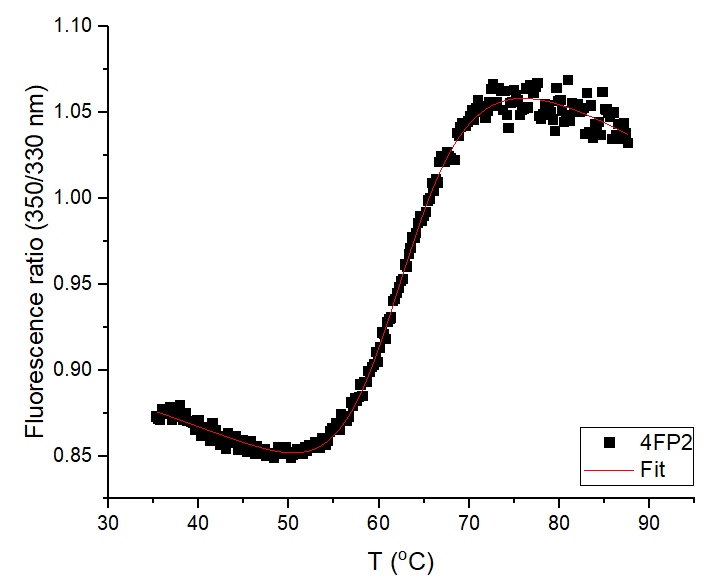
**TK-4F-Phe**


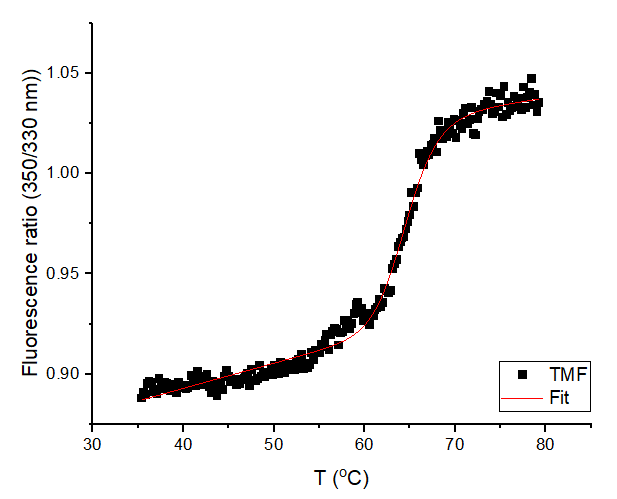
**TK-tfm-Phe**

**Figure S2. Fitting DSF data for thermal transition mid-points.** Triplicate sample data was pre-averaged prior to fitting.


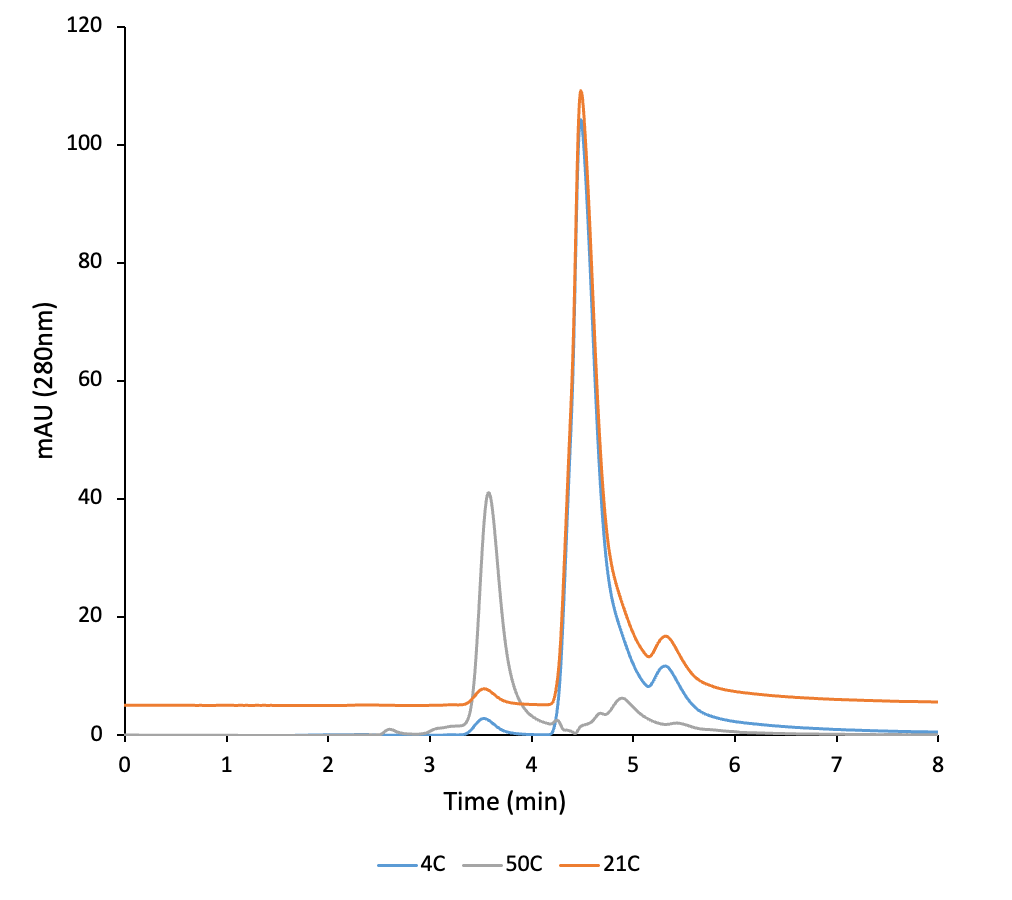
**WT-TK**


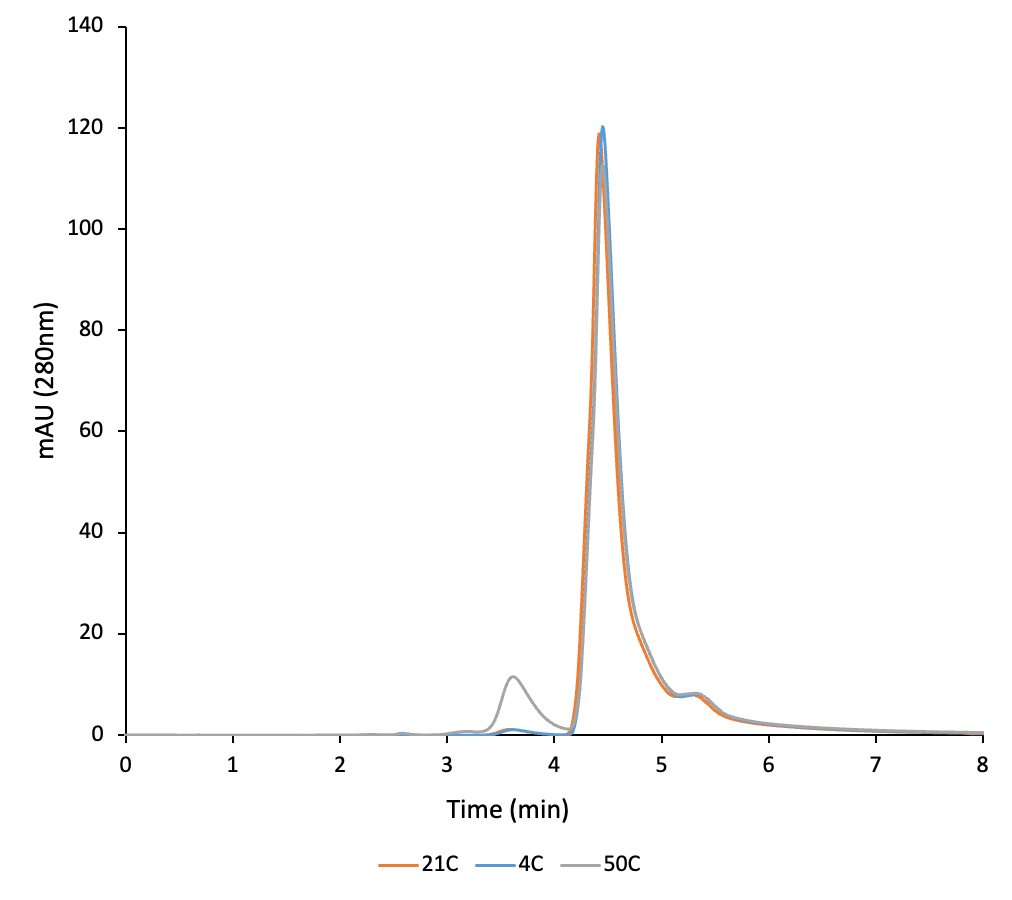
**TK-4F-Phe**


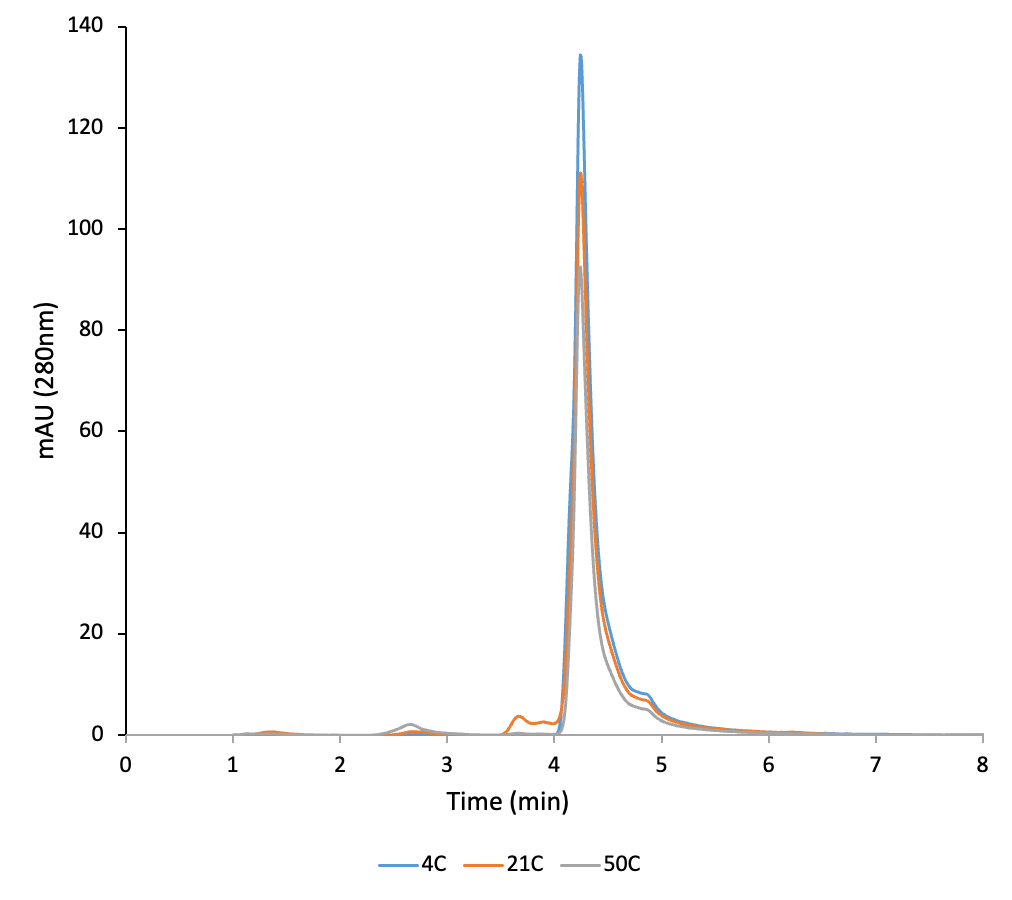
**TK-tfm-Phe**

**Figure S3. Size exclusion chromatography analysis.** 21 ^o^C trace for WT-TK has been offset by 5 mAU for visual clarity. Chromatograms are averaged from 3 to 6 repeat injections.


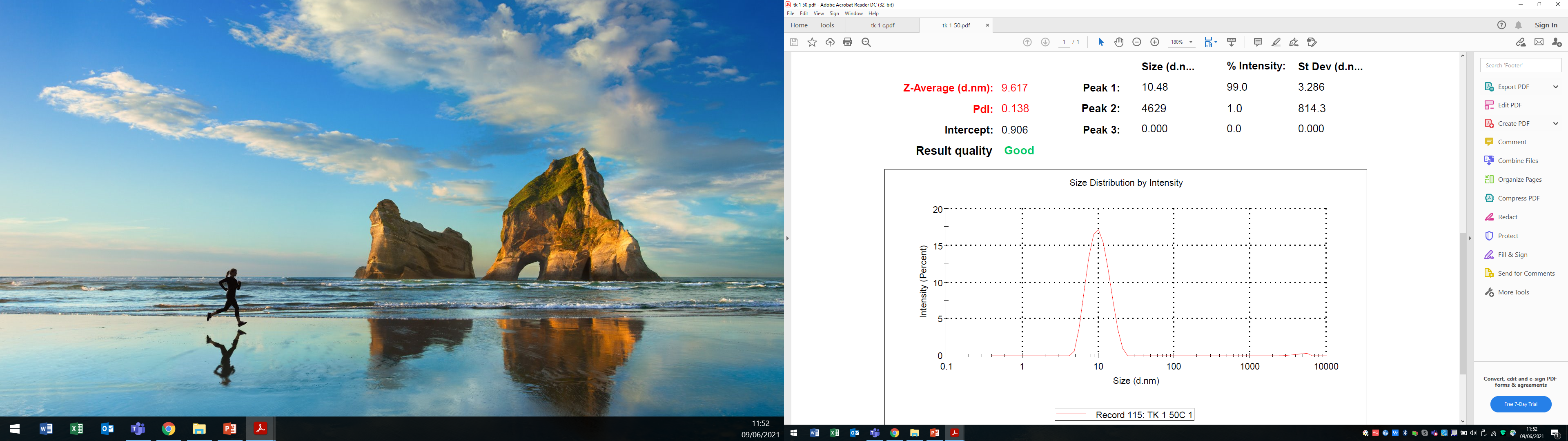
**A**


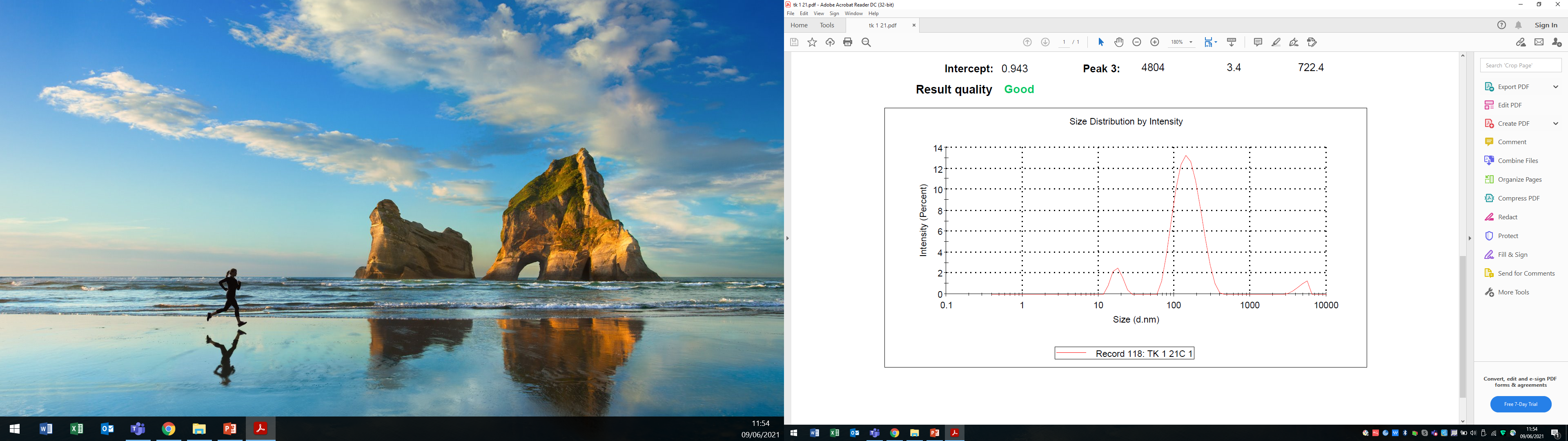
**B**

**Figure S4. Dynamic Light Scattering (DLS).**

DLS confirmed that heat treatment at 50 ^o^C led to the formation of larger aggregates. Sample shown is WT-TK. Each scattering curve was obtained from 10 repeated scans, and each in triplicate at 20 °C after A) 15 mins at 21 ^o^C, B) 15 mins at 50 ^o^C.


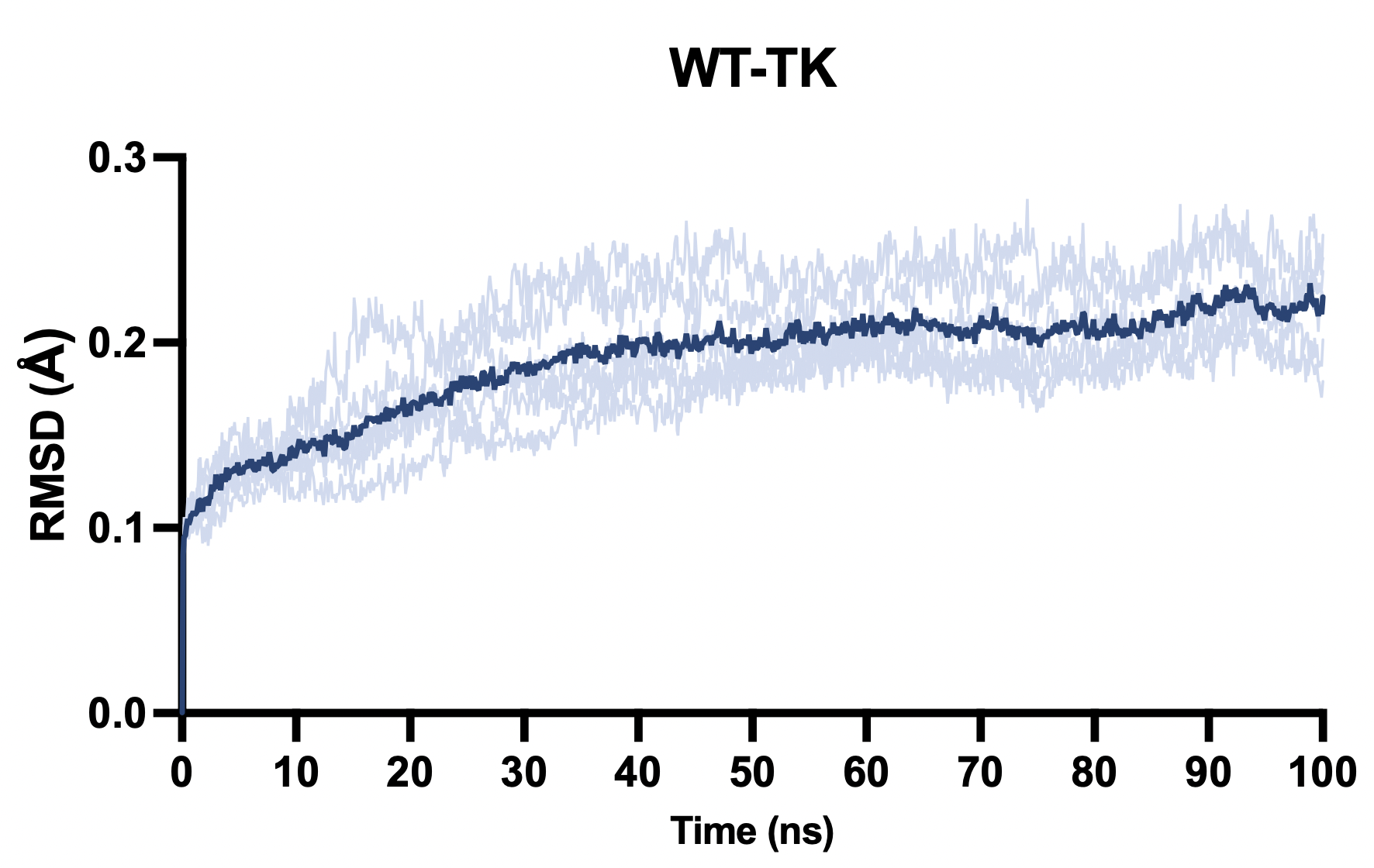


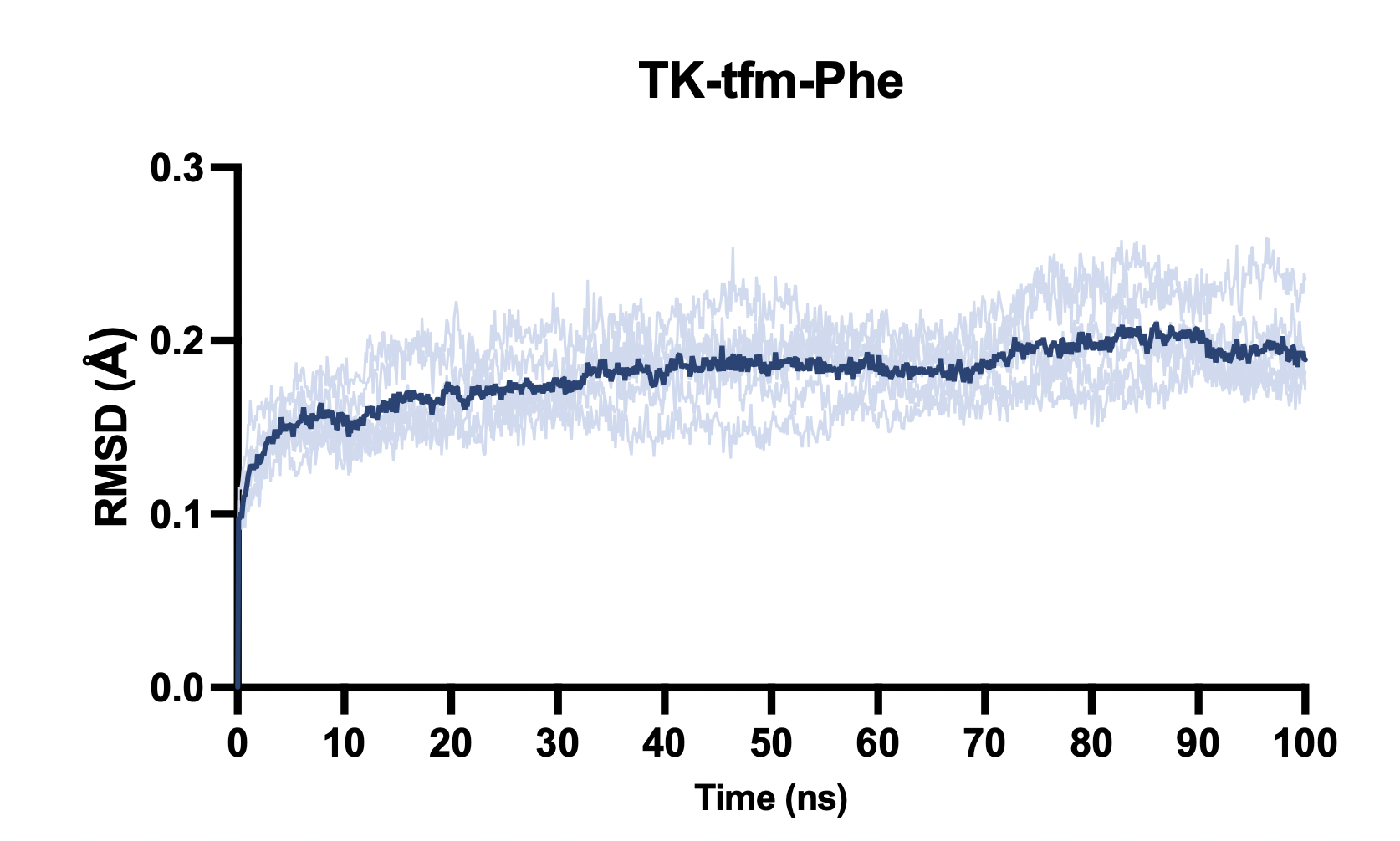


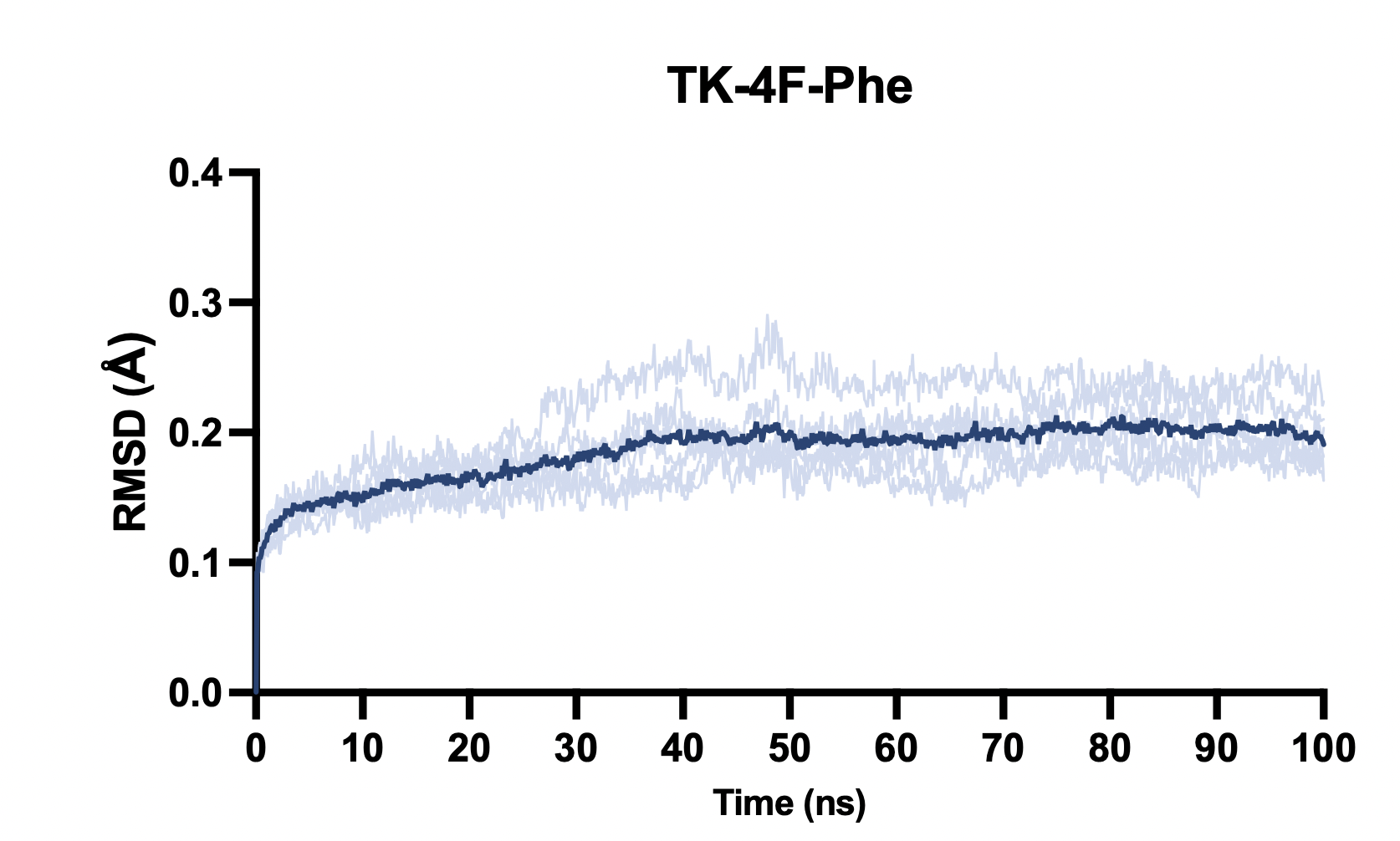


**Figure S5. RMSD versus simulation time for WT-TK and each variant.**

**
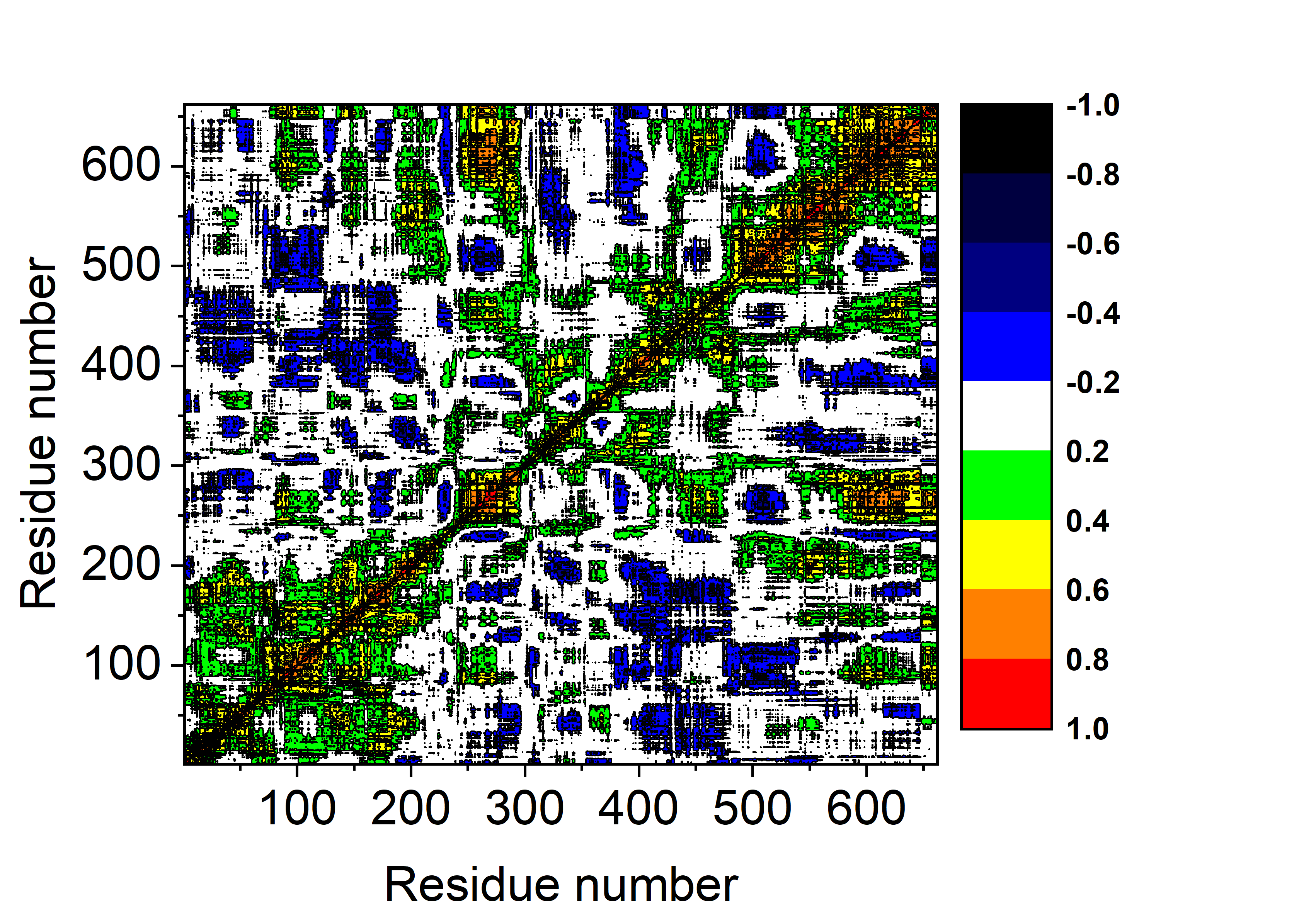

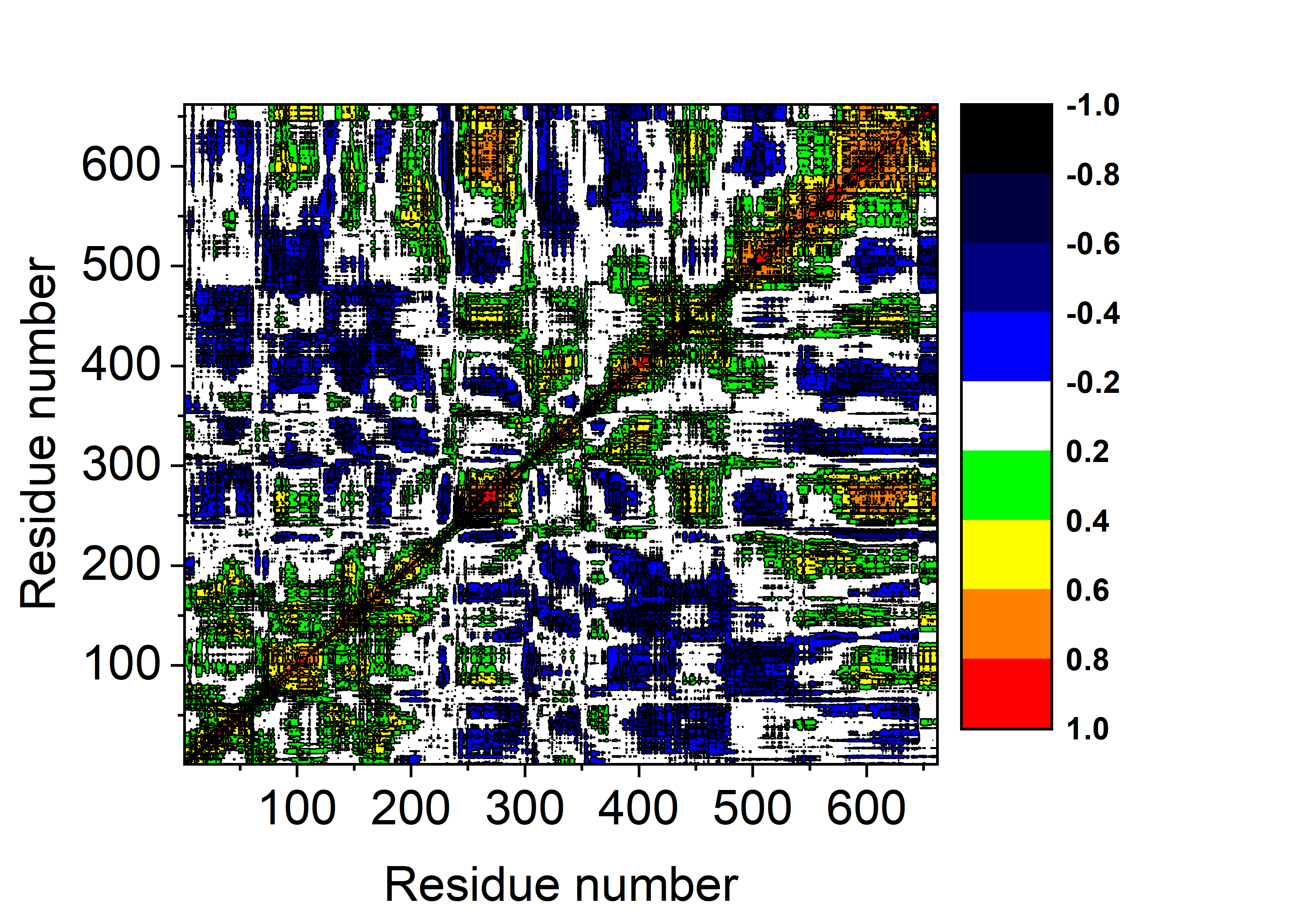
**

**WT tfm-Phe**


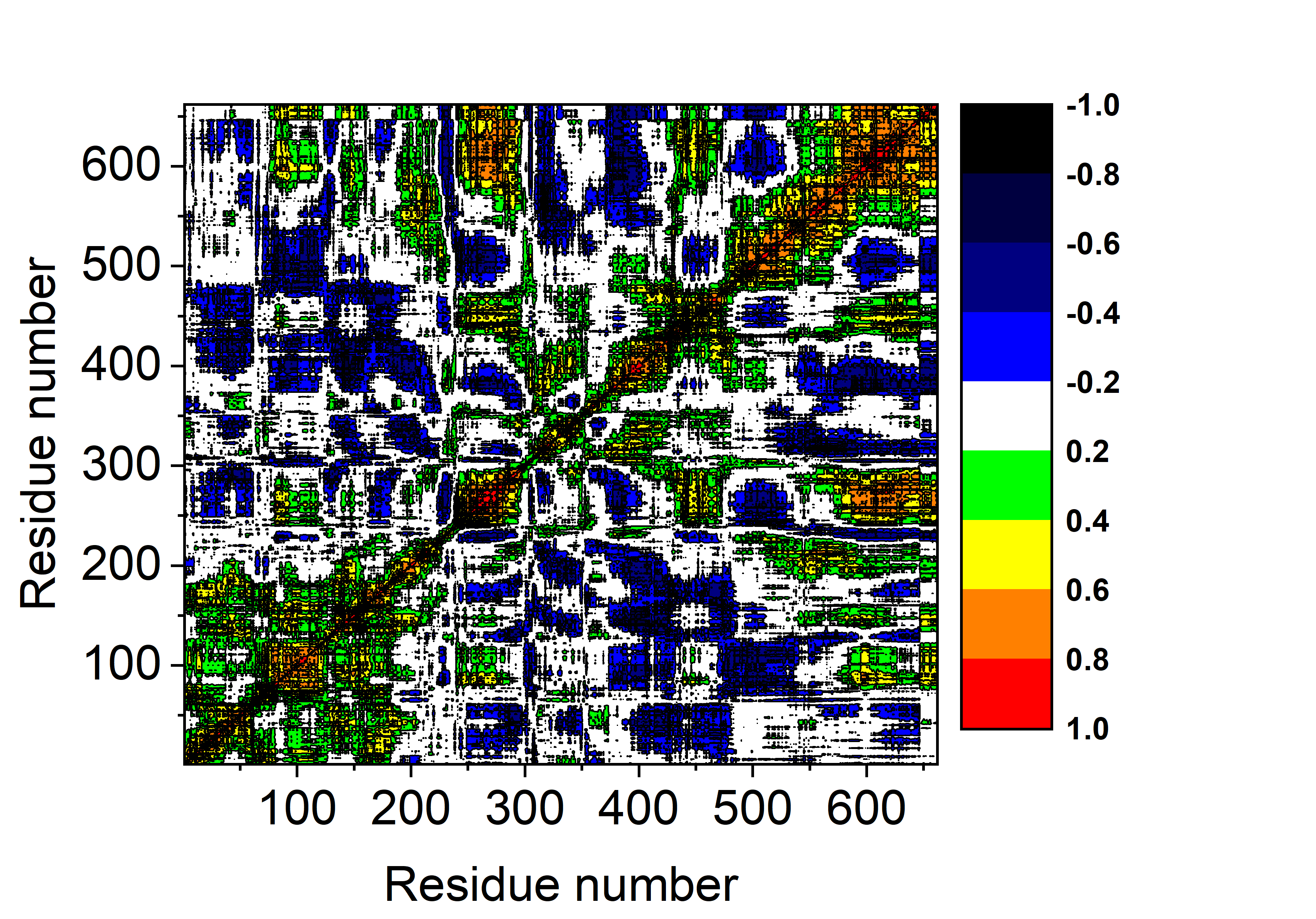


**4F-Phe**

**Figure S6. Dynamic cross-correlation matrices (DCCM) calculated from MD simulations at 300 K.** DCCM was calculated using frames from 40-100 ns and 6 repeats each, giving a total of 12 repeats by taking both chains from the homodimeric structure simulations. DCCM was calculated using Bio3D.
